# Protection of calves by a prefusion-stabilized bovine RSV F vaccine

**DOI:** 10.1038/s41541-017-0005-9

**Published:** 2017-03-08

**Authors:** Baoshan Zhang, Lei Chen, Chiara Silacci, Michelle Thom, Jeffrey C. Boyington, Aliaksandr Druz, M. Gordon Joyce, Efrain Guzman, Wing- Pui Kong, Yen-Ting Lai, Guillaume B. E. Stewart-Jones, Yaroslav Tsybovsky, Yongping Yang, Tongqing Zhou, Ulrich Baxa, John R. Mascola, Davide Corti, Antonio Lanzavecchia, Geraldine Taylor, Peter D. Kwong

**Affiliations:** 1 Vaccine Research Center, National Institute of Allergy and Infectious Diseases, National Institutes of Health, Bethesda, MD USA; 2 Institute for Research in Biomedicine, Università della Svizzera italiana, 6500 Bellinzona, Switzerland; 3 The Pirbright Institute, Ash Road, Pirbright, Woking, Surrey, GU24 0NF UK; 4 Electron Microscopy Laboratory, Cancer Research Technology Program, Leidos Biomedical Research, Inc., Frederick National Laboratory for Cancer Research, Frederick, MD USA; 5 Humabs BioMed SA, Via Mirasole 1, 6500 Bellinzona, Switzerland; 6 Institute for Microbiology, ETH Zurich, Wolfgang-Pauli-Strasse 10, 8093 Zurich, Switzerland

## Abstract

Bovine respiratory syncytial virus, a major cause of respiratory disease in calves, is closely related to human RSV, a leading cause of respiratory disease in infants. Recently, promising human RSV-vaccine candidates have been engineered that stabilize the metastable fusion (F) glycoprotein in its prefusion state; however, the absence of a relevant animal model for human RSV has complicated assessment of these vaccine candidates. Here, we use a combination of structure-based design, antigenic characterization, and X-ray crystallography to translate human RSV F stabilization into the bovine context. A “DS2” version of bovine respiratory syncytial virus F with subunits covalently fused, fusion peptide removed, and pre-fusion conformation stabilized by cavity-filling mutations and intra- and inter-protomer disulfides was recognized by pre-fusion-specific antibodies, AM14, D25, and MPE8, and elicited bovine respiratory syncytial virus-neutralizing titers in calves >100-fold higher than those elicited by post-fusion F. When challenged with a heterologous bovine respiratory syncytial virus, virus was not detected in nasal secretions nor in respiratory tract samples of DS2-immunized calves; by contrast bovine respiratory syncytial virus was detected in all post-fusion- and placebo-immunized calves. Our results demonstrate proof-of-concept that DS2-stabilized RSV F immunogens can induce highly protective immunity from RSV in a native host with implications for the efficacy of prefusion-stabilized F vaccines in humans and for the prevention of bovine respiratory syncytial virus in calves.

## Respiratory disease: A bovine model for respiratory syncytial virus vaccines

Researchers have produced a vaccine that protects against bovine respiratory syncytial virus (bRSV) in calves, with implications for humans. An international team comprising Geraldine Taylor, The Pirbright Institute, UK, Davide Corti and Antonio Lanzavecchia, Institute for Research in Biomedicine, Switzerland, and Peter Kwong, Vaccine Research Center, NIAID, NIH, United States, and their teams constructed the subunit vaccine from an engineered bRSVfusion (F) glycoprotein that protected challenged calves by generating a highly protective immune response. This approach allays some of the dangers of whole-virus vaccines. The results warrant further investigation, as current bRSV vaccines have significant downsides.Moreover, as the engineered bRSV F glycoprotein is structurally and reactively similar to the prefusion-stabilized human RSV (hRSV) F glycoprotein, the findings highlight potential benefits of similar vaccines in humans, as no licensed hRSV vaccine is currently available.

## Introduction

Bovine respiratory syncytial virus (bRSV) is a member of the *Paramyxoviridae* family that is responsible for the majority of respiratory disease in cattle annually, resulting in considerable morbidity and losses approaching $1 billion per year.^1–3^ bRSV is genetically and antigenically related to human RSV (hRSV),^2^ which is responsible for over 3 million hospitalizations for severe respiratory illness in young children and the elderly each year^4–6^ and for which no licensed vaccine is available. Although hRSV vaccines have been evaluated in mouse, cotton rat, and non-human primate (NHP) animal models, hRSV is only semipermissive in these animals and thus does not authentically represent natural infection. By contrast, bRSV infection in calves provides an opportunity to monitor RSV pathogenesis and RSV vaccine effectiveness in a natural host.^7^

Like infants, young calves are particularly vulnerable to bRSV, even in the presence of moderate levels of maternal antibodies,^8^ with prevalence rates of up to 70% in the first year of life.^3^ Although several licensed vaccines are available for bRSV, none are fully effective: low levels of maternal antibodies against bRSV can mitigate vaccine response in calves;^8, 9^ inactivated bRSV vaccines may enhance disease;^10, 11^ and live vaccines have the potential to exacerbate bRSV disease if administered intramuscularly in the presence of a concurrent bRSV infection.^12^ By contrast, recombinant subunit-based vaccines do not pose risks associated with live viruses, but do provide an opportunity to generate highly effective and targeted immune responses. They also have potential advantages in terms of ease and speed of manufacturing, quality control of purity, and long term stability.

**Fig. 1:**
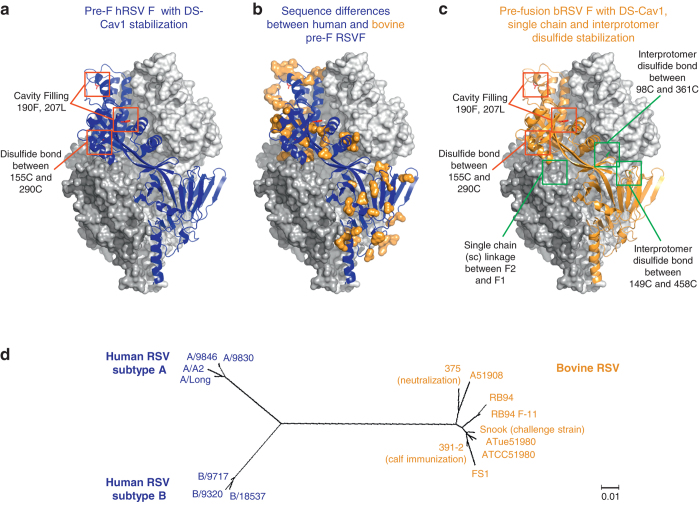
Translation of pre-F hRSV F stabilization to bRSV F. **a** Structural model of a pre-F hRSV F trimer stabilized by DS-Cav1 mutations (PDB ID 4MMU).^17^ One monomer is depicted by a *blue ribbon* model with the four DS-Cav1 mutations shown by *red stick* models outlined by *red squares*. The other two monomers are depicted by *gray surface* representations. **b** Sequence variation between hRSV strain A2 and eight different bRSV strains is mapped (*orange surface* representation) onto a *blue ribbon* model of one monomer of a DS-Cav1 pre-F RSV F trimer colored as in **a**. **c** The locations of the DS-Cav1 mutations (*red*), the sc linkage (*green*) and interprotomer disulfide stabilization mutations (*green*) introduced into bovine RSV F protein are indicated by *boxed stick models* for one RSV F monomer. In **b** and **c**, the other two monomers of the trimer are shown as *gray* surface representations. **d** Phylogenetic tree for human (*blue*) and bovine (*orange*) RSV F proteins. Names shown indicate the virus strain or isolate with study usage shown in parentheses. GenBank accession numbers for all strains used in this study are shown in Supplementary Table 1

The most potently neutralizing RSV antibodies identified thus far target the pre-fusion (pre-F) form of the RSV fusion (F) glycoprotein,^13, 14^ a type I fusion machine comprising a trimer of disulfide-bonded F_2_ and F_1_ heterodimers, which is responsible for virus entry and membrane fusion.^15, 16^ The pre-F form of RSV F is metastable and spontaneously undergoes structural rearrangements to the post-fusion (post-F) form, which no longer presents epitopes for many potently neutralizing antibodies. We recently employed structure-based design to engineer thermostable versions of the pre-F hRSV F glycoprotein,^17, 18^ which preserve the pre-F conformation and the associated target epitopes for highly potent neutralizing monoclonal antibodies (mAbs) such as AM14 and D25 (refs.^19^, ^20^). These immunogens were subsequently observed to elicit high levels of neutralizing antibodies in immunized mice and NHPs.^17, 18^ Since the F glycoprotein of bRSV has greater than 80% sequence identity with that of hRSV^2^ (Supplementary Fig. 1), we hypothesized that bRSV F could be stabilized in an analogous way to create a similarly potent bRSV vaccine. We, therefore, transferred the hRSV F mutations referred to as DS-Cav1 (ref. 17), single chain (sc) alterations (sc9 and sc9-10) (ref. 18), and interprotomer disulfides (Q98C Q361C, A149C Y458C, and N183GC N428C)^18^ to equivalent positions in multiple strains of bRSV to create bRSV F trimer immunogens stabilized in the pre-F state (Fig. 1a–c). Evaluation of the immunogenicity of these immunogens in both mice and calves resulted in high-titer neutralizing responses, with heterologous bRSV challenge in calves revealing protection from viral replication, lung inflammation, and clinical signs of disease—with no evidence of vaccine-associated disease enhancement—an important milestone in the development of an effective bRSV subunit vaccine. In addition, the high titer protective response elicited by the prefusion-stabilized F in calves bodes well for the elicitation of similar high titer response in humans immunized with similarly stabilized F immunogens.

## Results

### Design and initial characterization of bRSV F immunogens

The RSV F glycoprotein is conserved between bRSV and hRSV, with sequence identities of ~80% (ref. 2) (Fig. 1b, d, Supplementary Fig. 1 and Supplementary Table 1), and multiple F-directed antibodies can neutralize both hRSV and bRSV.^21–24^ Based on the success of our prior engineering of pre-F hRSV F trimer,^17^ we modified bRSV F to create thermostable pre-F trimers. A disulfide between residues 155 and 290 (DS) along with cavity-filling mutations S190F and V207L (Cav1) and a C-terminal T4-phage fibritin trimerization domain (foldon) were incorporated into bRSV F from seven different strains to make bovine versions of DS-Cav1 (ref. 17) (bDS-Cav1s) (Fig. 1a, b and Supplementary Table 2), which included cleavable C-terminal His and Strep tags for purification. Upon expression in Expi293F cells only three of the seven bDS-Cav1s (strains 391-2, ATue51908, and RB94, respectively) expressed at greater than 0.5 mg/l of culture (Supplementary Table 2). All three of these bDS-Cav1s were recognized by pre-F-specific mAbs D25 (ref. 20) and MPE8 (ref. 21) as well as by mAb motavizumab (Mz)^25^ (Supplementary Table 2).

To enhance immunogenicity, we next sought to optimize bDS-Cav1 thermostability. To minimize the number of designs evaluated, we selected two RSV strains (391-2 and RB94) to optimize initially, with the intent to introduce the best mutations from the final set into the third strain, ATue51908. Previous investigations^18, 26^^–^^29^ of type I fusion machines have indicated that removal of the cleavage site to create sc variants can improve pre-F stability. Furthermore, the introduction of interprotomer disulfide bonds (DS2) has been observed to enhance both stability and immunogenicity of hRSV F immunogens.^18^ Therefore, with a focus on these two aforementioned types of stabilizations, 90 variants of bDS-Cav1 were designed, all of which employed an sc topology and 32 of which contained an interprotomer disulfide (DS2 variants). Additionally, many of the 90 designs incorporated internal cavity-filling mutations, core residues from hRSV F for increased stability, and additional sites of *N*-linked glycosylation to mask irrelevant epitopes.

All 90 bDS-Cav1 designs were evaluated for expression and antigenic recognition by mAbs D25, MPE8 and Mz in a 96 well-microplate transient transfection format.^17^ Each design was scored by summing enzyme-linked immunosorbent assay (ELISA) readings for the pre-F-specific mAbs D25 and MPE8 (Supplementary Tables 3 and 4). The top three-scoring DS2 designs (DS2-v1, DS2-v5, and DS2-v3) and the top two-scoring 391-2 and RB94 sc designs without interprotomer disulfides (sc-v1 and sc-v4) were selected for additional evaluation (Supplementary Tables 3 and 4). To expand the dimensions of our search for optimal immunogens, we mixed and matched DS2 interprotomer disulfides (Q98C Q361C, A149C Y458C, and N183GC N428C) and sc formats (sc9 and sc9-10) from the top five-scoring designs and added the ATue51908 strain to generate additional designs for a total of nine constructs, which were expressed in 1 liter Expi293F cultures (Supplementary Table 5). The sc designs sc9 and sc9-10 differed only in two residues, with a GS linker replacing F_2_F_1_ sites of cleavage and fusion residues 106–144 or 104–144, respectively.^18^ Of these nine designs, the two sc-only variants gave 7–9-fold higher expression yields compared to the other variants (Supplementary Table 5). However, size exclusion chromatography (SEC) indicated their respective molecular sizes were larger than expected, suggesting unfolding or aggregation. We, therefore, chose three of the remaining five DS2 designs (each with sc topology and added interprotomer disulfides) with the highest yield (Supplementary Table 5) for immunogenic, antigenic, physical, and structural characterizations. Additionally, as benchmarks, we used the DS-Cav1 variant of each of the three strains and also the post-F form of each of the three strains (the latter created by removing the RSV F fusion loop residues 137–146), as previously described.^30^ Altogether, the sc-DS2, DS-Cav1 and post-F immunogens of each of the three strains totaled nine final immunogen constructs.

All nine of these constructs gave expression yields of 2–5 mg/l except for DS2-v35 (0.24 mg/l) and RB94 DS-Cav1 (0.76 mg/l) (Table 1), and the post-F forms consistently gave the highest expression (3–5 mg/l). After purification on nickel and Strep-Tactin affinity columns, and subsequent cleavage of C-terminal affinity tags, all nine immunogens behaved well when analyzed by SEC. Post-F forms eluted at slightly larger sizes than pre-F forms due to their elongated shape,^20, 30^ and pre-F forms all eluted with peaks consistent with trimer formation (Supplementary Fig. 2a, b).

**Table 1:** Antigenic and physical characterization of bRSV F glycoprotein immunogens

Variant number	bRSV F variant	Yield (mg/l)^a^	Antibody affinity *K*_D_ (nM)^b^				Physical stability (fractional antibody reactivity^c^)						
				QP^d^			Temperature (°C)		pH		Osmolarity (mM)		Freeze-thaw
			Site Ø	Site III	Site V	Site II							
			D25	MPE8	AM14	Mz^e^	50	70	3.5	10.0	10	3000	10^f^
DS2-v1	391-2 sc9 DS-Cav1 Q98C Q361C	2.80	24.3	0.4	8.6	0.8	0.9	0.8	1.2	1.0	1.0	0.9	1.0
DS2-v33	391-2 sc9-10 DS-Cav1 Q98C Q361C	2.44	57.5	0.8	12.8	1.2	1.0	1.0	0.9	0.9	0.8	0.8	1.0
–	391-2 DS-Cav1	3.66	0.4	0.2	1.8	0.8	0.7	0.1	0.9	1.2	0.9	0.6	1.0
–	391-2 post-F	4.10	>1000	>1000	>1000	0.6	1.0	1.0	0.8	1.1	0.9	0.6	1.0
–	ATue51908 DS-Cav1	2.98	10.8	0.2	4.8	0.8	0.9	0.1	0.9	1.1	0.7	0.4	1.0
DS2-v35	ATue51908 sc9-10 DS-Cav1 A149C Y458C	0.24	420	0.5	12.8	1.2	0.9	0.9	1.0	0.9	0.7	0.8	1.0
–	ATue51908 post-F	3.80	>1000	>1000	>1000	0.5	1.0	1.0	0.9	1.1	1.0	0.8	1.0
–	RB94 DS-Cav1	0.76	24.3	0.2	4.9	0.9	0.6	0.1	1.2	1.3	0.8	0.5	1.0
–	RB94 post-F	4.78	>1000	>1000	>1000	0.4	1.0	1.0	0.9	1.1	1.0	1.0	1.0

The properties of the trimer fraction purified by gel filtration are listed

^a^Yield reported is only for the trimer fraction

^b^When no binding was observed for 1 μM Fab, the *K*_D_ is shown as >1000

^c^D25 was used for pre-F proteins and Mz was used for post-F proteins

^d^QP: quaternary-specific antibody sites

^e^Motavizumab (Mz)

^f^Ten cycles of freeze-thaw in the presence of 10% glycerol

### Antigenic characteristics of bRSV F immunogens

The antigenicity of each purified immunogen was evaluated with biolayer interferometry to assess recognition by the antigenic site Ø-directed mAb D25 (ref. 20), antigenic site II-directed mAb Mz^25, 31^ and quaternary-specific mAbs AM14 (ref. 19) and MPE8 (ref. 21) (Table 1). The three pre-F-specific mAbs, D25, MPE8, and AM14, recognized all six immunogens containing pre-F stabilizing mutations, confirming stabilization of their pre-F conformations. Moreover, recognition by the quaternary-specific mAbs MPE8 and AM14 substantiated the formation of native-like trimers. In contrast, these mAbs did not recognize any of the post-F immunogens. As expected, Mz recognized all nine immunogens since its site II epitope is not affected by pre- and post-F conformational changes. D25 recognized the pre-F immunogens with affinities ranging from 0.4–420 nM, suggesting that antigenic site Ø may be adversely affected by some of the stabilizing mutations. The three DS-Cav1-only immunogens which also had the least number of mutations had the highest affinity for D25. Notably, all mAbs except for D25 recognized the pre-F immunogens with nanomolar affinity (0.2–12.8 nM) even though these mAbs were elicited by hRSV.

### Physical characteristics of bRSV F immunogens

We next assessed the stability of purified immunogens by subjecting them to extremes of temperature, pH, and osmolarity as well as cycles of freeze-thaw and quantifying their subsequent recognition by D25 (for pre-F) or Mz (for post-F) (Table 1). All nine immunogens were observed to generally tolerate pH and osmolarity extremes and freeze-thaw cycles, consistent with data reported for hRSV DS-Cav1 (ref. 17). The three DS-Cav1-only immunogens, 391-2 DS-Cav1, ATue51908 DS-Cav1 and RB94 DS-Cav1, were most susceptible to physical extremes and lost 25–60% of their D25 reactivity upon exposure to high (3M) osmolarity. Curiously, the majority of immunogens actually increased reactivity to D25 or Mz after exposure to high pH. Not surprisingly, all three post-F immunogens, were stable at higher temperatures as measured by Mz affinity. Although none of the DS-Cav1-only immunogens were able to antigenically survive exposure to 70 °C, consistent with that observed for hRSV F pre-F-stabilized immunogens,^17, 18^ all three of the DS2 immunogens tolerated exposure to high temperature.

### Structural characteristics of bRSV F immunogens

To further confirm the pre- and post-F conformations of the engineered bRSV F immunogens, we examined them by negative stain electron microscopy (EM), followed by reference-free two-dimensional (2D) class averaging of the images (Supplementary Fig. 2c). As expected, each of the pre-F immunogens displayed bulb-like trimer structures with a short stem-like structure at one end, whereas the post-F immunogens displayed longer and more slender structures, each of which was consistent with known crystal structures of pre- and post-F hRSV F.^17, 20, 30, 32^

**Fig. 2:**
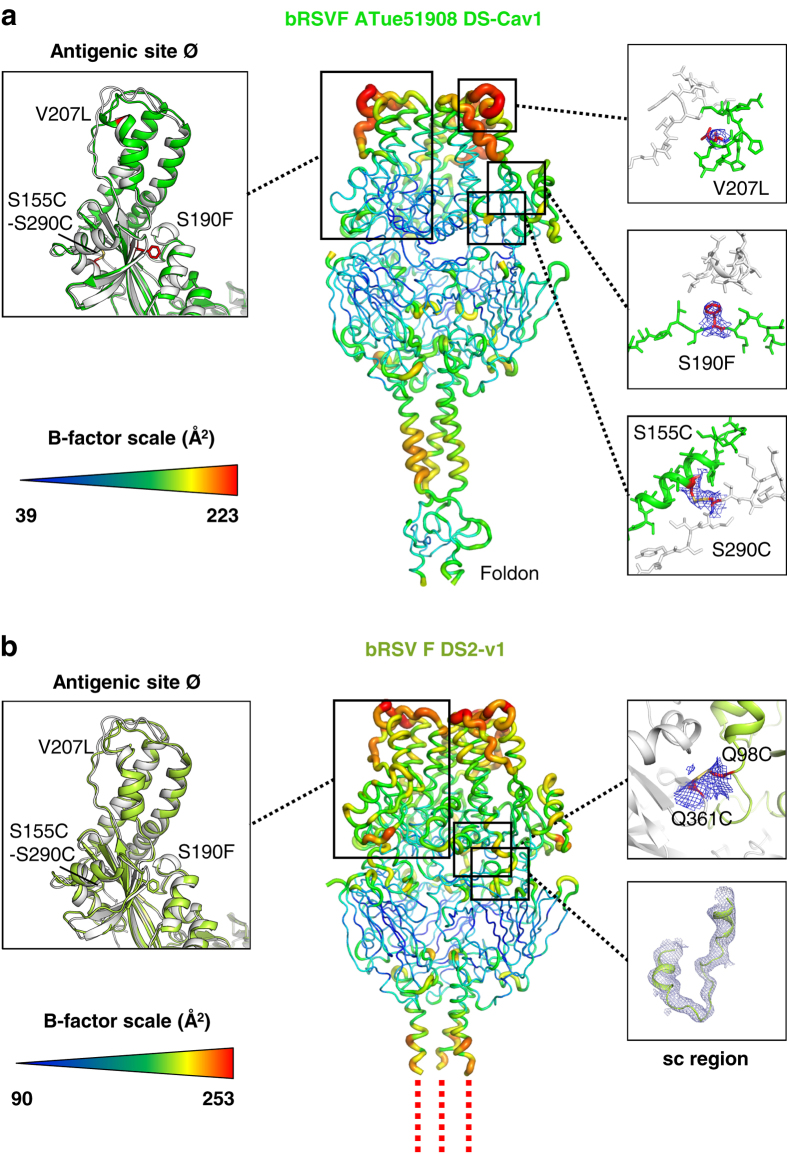
Crystal structures of pre-F-stabilized bRSV F immunogens. **a** Crystal structure of bRSV F ATue51908 DS-Cav1 depicted by a Cα-worm representation color-coded by atomic mobility factors, with *thick*, red worm for *flexible* regions and *thin*, blue worm for more *rigid* regions. Atomic level details are shown in *insets* on the right with stick representations and *2Fo-Fc* electron density (*blue*) for regions that were mutated to stabilize the pre-F conformation. The upper left *inset* shows a ribbon superposition of the antigenic site Ø region of ATue51908 DS-Cav1 (lime) with the structure of hRSV F DS-Cav1 (*gray*; PDB ID 4MMU).^17^ **b** Crystal structure of the DS2 immunogen bRSV F DS2-v1, depicted as in **a**

The crystal structures of two pre-F-stabilized bRSV F immunogens, ATue51908 DS-Cav1 and DS2-v1 were determined to 2.65 and 3.50 Å resolution, respectively (Supplementary table 6). ATue51908 DS-Cav1 crystallized in a monoclinic lattice not previously observed with hRSV F immunogens. The overall structure of ATue51908 DS-Cav1 was similar to that of hRSV DS-Cav1 (PDB ID 4MMU)^17^ with a root mean square deviation (rmsd) of 1.0 Å for 437 Cα atoms excluding residues 209–215 in a membrane distal loop adjacent to antigenic site Ø (Fig. 2a). In this crystal form, the appended C-terminal foldon trimerization domain was visible in the electron density map, although its threefold axis was tilted by ~17 degrees relative to the threefold axis of bRSV pre-F due to crystal packing (Fig. 2a). The introduced DS and S190F mutations showed strong electron density, while V207L showed weaker but detectable electron density (Fig. 2a). The DS2 immunogen DS2-v1 crystallized in the cubic lattice commonly observed with hRSV F pre-F immunogens and, like ATue51908 DS-Cav1, its structure was similar to hRSV DS-Cav1 with an rmsd of 1.1 Å for 435 Cα atoms. Although side chains for the DS-Cav1 mutations were not clearly apparent in the electron density, partly due to the 3.50 Å resolution of the structure, the DS2 interprotomer 98C-361C disulfide and nearby sc linker showed traceable electron density (Fig. 2b). We observed the 98C-361C disulfide bond to cause a local distortion of the α1 helix (in which 98C is located), although the local structure surrounding 361C was unperturbed. Comparison of the two bRSV pre-F-stabilized structures at the backbone level revealed high-structural similarity with an rmsd of 0.9 Å between 434 equivalent Cα atoms excluding the F_2_F_1_ linker region. The greatest structural differences between the hRSV and bRSV pre-F immunogens were observed in residues 206–215 at the apical loop between α4 and α5 near antigenic site Ø (Fig.2a, b, left panels), which is also the region of highest sequence divergence (only ~50% identity) between the two species (Supplementary Fig. 1). These structural and sequence difference may explain the lower-binding affinity of D25 to bRSV pre-F (Table 1) relative to hRSV pre-F.^17^ Overall, the structural analysis confirmed the similarity of bRSV and hRSV pre-F structures, with subtle differences in specific regions, including a loop near antigenic site Ø.

### Immunogenic characterization of bRSV F immunogens in mice

**Fig. 3:**
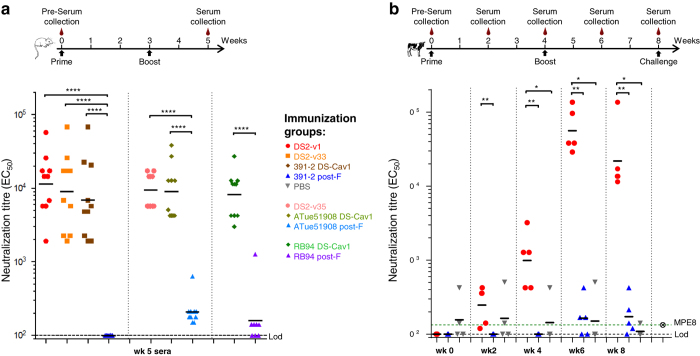
Serum neutralizing antibody titers elicited by engineered bRSV F pre-F trimers. Pre-F-stabilized bRSV F glycoproteins elicited geometric mean EC_50_ neutralization titers between 43–344-fold higher than post-F in mice and calves, respectively. Schematic immunization procedures for bRSV F variants in seronegative mice (**a**) and calves (**b**). Neutralization titer from each animal is shown as an individual *dot*, and geometric means are indicated by *black horizontal lines*. Immunization groups are color-coded. Lod, limit of detection (titer = 100) is indicated with a *horizontal dashed line*. *Vertical dotted lines* separate immunogen strains in **a** and weeks post prime in **b**. Serum antibody binding ELISA data is summarized in Supplementary Fig. 3. *P* values were determined by two-tailed Mann–Whitney tests. *Indicates *P* ≤ 0.05, **indicates *P* ≤ 0.01, ***indicates *P* ≤ 0.001 and ****indicates *P* ≤ 0.0001. There are 10 mice per group for the mouse immunizations. For calf immunizations, the DS2-v1 and post-F groups each contained five animals and the placebo group contained four animals

To evaluate immunogenicity, each of the nine bRSV F immunogens was used to immunize a group of 10 CB6F1/J mice. Each immunogen dose comprised 10 μg of protein adjuvanted with 50 μg of polyinosinic:polycytidylic acid (Poly I:C). Mice were primed and boosted intramuscularly at weeks 0 and 3, respectively. Analysis of week 5 sera revealed geometric mean reciprocal EC_50_ neutralization titers of 6880–11,453 for pre-F immunogen-immunized mice, which were 33- to 55-fold higher (*P* < 0.0001) than the titers (geometric mean 100–210) observed for the post-F immunogen-immunized mice (Fig. 3a and Supplementary Table 7). Neutralization titers elicited from pre-F-immunized mice were 82–110-fold greater than the calibrated protective titer of 100 (ref. 17). Although DS2-v1 elicited the highest titers (geometric mean 11,453), all of the pre-F immunogen-elicited titers were statistically comparable with each other. Thus the 1000-fold difference in D25 mAb-binding affinities observed between various pre-F immunogens (Table 1) did not appear to impact the neutralization titers of elicited sera. To gauge the overall immunogenicity of each immunogen, the sera binding response to pre-F and post-F immunogens was assessed by ELISA (Supplementary Fig. 3a). Similar to the neutralization results, the binding titers of pre-F elicited sera to the six pre-F immunogens were statistically comparable to each other with geometric mean end point-binding titers ranging from 4687 to 100,323. Intriguingly, sera from DS2-v1-immunized mice displayed the lowest titers for pre-F immunogen even though it had the highest titers of neutralizing antibodies. As expected, sera elicited by pre-F immunogens displayed lower-binding titers to post-F immunogens.

### Immunogenic characterization of bRSV F immunogens in calves

To investigate the effectiveness of pre-F-stabilized RSV F vaccines in bRSV-seronegative calves, we selected the highly stable DS2 immunogen (DS2-v1), which in mice elicited the highest neutralization titers (geometric mean reciprocal EC_50_ 11,453). As controls we chose post-F 391-2 and used phosphate-buffered saline (PBS) to immunize a placebo group. Groups of five 3–6-week-old male calves (Supplementary Table 8) were immunized twice at weeks 0 and 4, and sera were collected 2 weeks after each immunization. Each injection consisted of 50 μg protein in 0.6 ml PBS adjuvanted with 1.4 ml of an oil-in-water adjuvant ISA 71G. The reciprocal EC_50_ neutralization titers from the DS2-immunized calves were observed to increase exponentially at weeks 2, 4, and 6 relative to week 0, resulting in a final geometric mean titer of 56,055 2 weeks after the boost (Fig. 3b and Supplementary Table 9). At week 8, 4 weeks after the boost, titers dropped to a geometric mean of 21,849. In contrast, by week 8, post-F immunogen elicited minimal titers (geometric mean 172) within the same range as two of the saline control-immunized calves (141 and 505, respectively), which may have had maternally-derived serum antibodies at the start of the study. Results from ELISA analysis of sera from DS2-immunized calves mirrored the neutralization titers with binding titers steadily increasing and then slightly decreasing from weeks 6 to 8 (Supplementary Fig. 3b). As expected, sera from post-F-immunized calves followed a similar trend, reaching geometric mean titers approximately 3.0 and 3.8-fold lower than DS2 by weeks 6 and 8, respectively. It is also clear from the neutralization data (Fig. 3b) that post-F elicited significantly lower levels of neutralizing antibodies. Not surprisingly, sera elicited by DS2 immunogen displayed lower-binding titers to post-F immunogen, and post-F-elicited sera recognized both pre- and post-F with comparable titers (as observed with hRSV DS-Cav1 immunization in NHPs).^17^ Results from a competition ELISA showed that the week 6 sera from DS2-immunized calves competed with the broadly neutralizing mAbs AM14, D25, RSD5 (ref. 21), MPE8 and palivizumab (Pz),^25^ suggesting that antigenic sites Ø, II, III, and V were all targeted^33^ (Supplementary Fig. 4). Although the mAb Mz showed considerably less competition than the other antigenic site II mAb, Pz, its high 34.6 pM affinity^25^ for RSV F likely limited sera competition. As expected, sera from post-F-immunized calves competed to a much lower extent with the pre-F-specific mAbs, AM14, RSD5, and MPE8, and did not compete with D25. Interestingly, two mAbs compatible with post-F RSV F, Mz and Pz, also competed to a much lower extent with post-F-elicited sera. This suggests that a minority of the post-F-elicited sera was directed against site II. Overall, the results in calves demonstrate the striking superiority of the pre-F stabilized DS2-immunogen vs. post-fusion F, with pre-F-induced neutralizing titers more than 100-fold higher than post-F-induced neutralizing titers by week 6.

### bRSV challenge of immunized calves

**Fig. 4:**
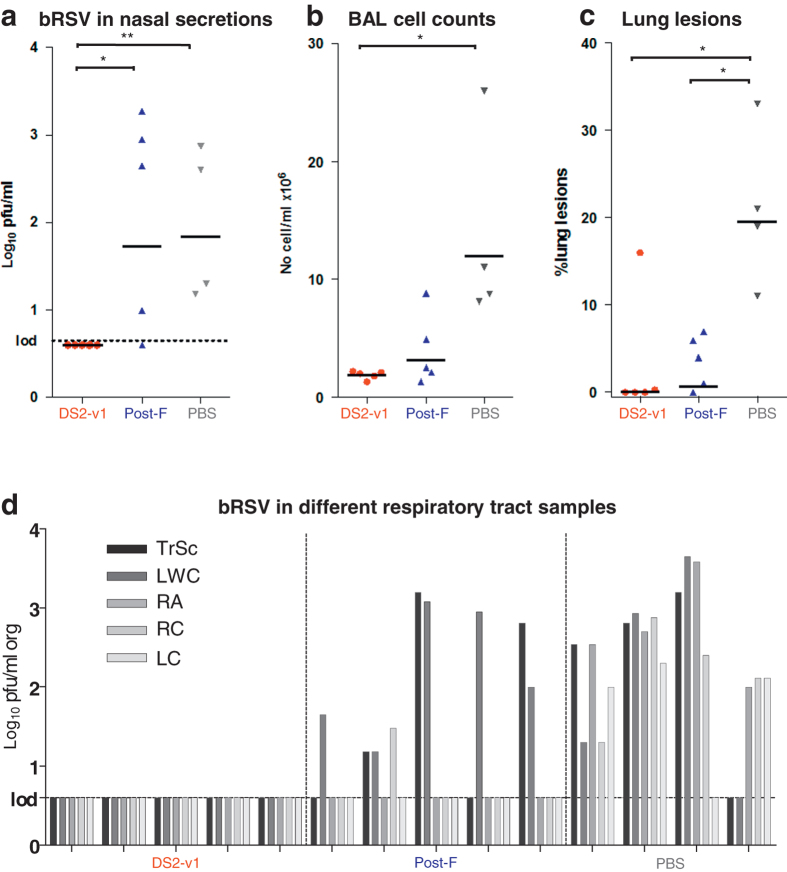
Effect of vaccination on bRSV replication in the respiratory tract of calves and on pulmonary pathology. **a** Peak titers of bRSV in nasal secretions. Each dot represents the virus titer from nasopharyngeal swabs obtained at day 6 post challenge. Groups of five calves were vaccinated with DS2-v1, Post-F (391-2 post-F), or PBS (Placebo in adjuvant). Geometric mean peak titers are indicated by *black horizontal lines*. **b** Effect of F protein vaccination on numbers of cells in BAL, 6 days after challenge with bRSV. **c** Analysis of percentage of lung with macroscopic lung lesions from photographs of lungs. **d** bRSV titers in samples of tracheal epithelium (TrSc), lung wash cells (LWC), and homogenates of samples taken from the right apical (RA), right cardiac (RC), and left cardiac (LC) lobes of the lung, 6 days post-infection. Each bar represents the bRSV titer of a lung sample. Each group of five bars is from an individual calf. Titers are expressed as log_10_ pfu/ml or g. The limit of detection (lod) is log_10_ 0.7 pfu/ml or g (**a** and **d**). Virus titers for each individual testing point are listed in Supplemental Table 8. *P* values were determined by two-tailed Mann–Whitney tests. *Indicates *P* ≤ 0.05, **indicates *P* ≤ 0.01, ***indicates *P* ≤ 0.001 and ****indicates *P* ≤ 0.0001

Next, all calves were challenged by intranasal and intratracheal routes with the heterologous Snook strain of bRSV, 4 weeks after the boost. Calves were monitored daily for clinical signs of disease and for viral titers in the nasopharynx for 6 days after challenge. At day 6 after challenge, calves were euthanized and bronchoalveolar lavage (BAL) and lung biopsies from three regions of the lung were obtained to determine viral titers, neutrophil infiltration, and the extent of microscopic and macroscopic lesions. Remarkably, calves vaccinated with DS2 had no detectable bRSV viral titers in nasopharyngeal secretions (Fig. 4a and Supplementary Table 10). No detectable bRSV titers were observed from a postmortem lung wash, samples of tracheal epithelium, right apical, right cardiac or left cardiac regions of the lung (Fig. 3d and Supplementary Table 11). In contrast, peak nasopharyngeal virus titers 6 days post challenge ranged from 1.81 to 2.70 log_10_ pfu/ml in post-F and PBS vaccinated calves (Supplementary Table 10). Likewise, virus was isolated post mortem from BAL cells of all of the post-F-immunized and PBS-immunized calves with the greatest extent of lung virus replication in the PBS-immunized control animals (Fig. 4d). Thus, all DS2-immunized calves were protected from bRSV viral replication in both the upper and lower respiratory tracts. Furthermore, four out of five of the DS2-immunized calves were also protected from clinical signs of disease, lung inflammation and macroscopic lung lesions (Fig. 4b, c, Supplementary Fig. 5 and Supplementary Tables 12–14). Clinical scores, based mainly on differences in respiratory rate (RR) and body temperature, were minimal for most of the pre-F- and post-F-immunized calves. Scores trended higher for the PBS-immunized controls, but were not significantly different from the other two groups (Supplementary Tables 12–13). However, both RR and body temperature increased in all PBS-immunized calves, 6 days after bRSV challenge, whereas the RR increased in only two post-F-immunized and one pre-F-immunized calves at this time (Supplementary Fig. 5a). Although the one calf in the pre-F-immunized group that had developed a raised RR and body temperature also exhibited signs of lung inflammation, the geometric mean number of cells observed in the BAL, the percentage of polymorphonuclear neutrophils (PMNs) in BAL, and the percentage of macroscopic lung lesions were all statistically lower than in the placebo group (Fig. 4b, c and Supplementary Tables 12–14). The post-F-immunized calves displayed intermediate levels of lung inflammation. Although the extent of macroscopic lung lesions in the post-F-immunized calves was less than that observed in the placebo group, the percentage of PMNs in BAL was similar to that seen in BAL from calves in the placebo group. Microscopic lung lesions in the placebo group, 6 days post challenge, were typical of bRSV bronchiolitis and alveolitis and were characterized by epithelial necrosis and hypertrophy of bronchiolar epithelium bronchiolitis, bronchiolar exudate containing desquamated epithelial cells, neutrophils and macrophages, and thickening of alveolar walls due to infiltration by mononuclear cells and granulocytes (Supplementary Fig. 6c). In addition to bronchiolitis and alveolitis, peribronchiolar lymphoreticular hyperplasia were seen in three of the post-F-immunized calves (Supplementary Fig. 6b). In contrast, bronchiolitis and alveolitis were absent from all but one of the DS2-immunized calves, and the histopathology was essentially restricted to a peribronchiolar lymphoreticular hyperplasia (Supplementary Fig. 6a).

## Discussion

The development of safe and effective hRSV and bRSV vaccines continues to be a priority.^2, 15, 34^ For both human infants and calves, vaccines need to surmount similar hurdles of activating very young immune systems and overcoming vaccine inhibition due to the presence of maternal antibodies. Currently, there is no licensed hRSV vaccine, and bRSV vaccines continue to suffer from issues of effectiveness.^3^ The antigenic and genetic similarities between hRSV and bRSV and the need for an authentic animal model for hRSV present a unique opportunity to evaluate stabilized pre-F vaccine effectiveness for both diseases in a bovine animal model. Unlike the semipermissive rodent and NHP animal models for hRSV currently in use, bRSV infects the bovine host and engages the innate and adaptive immune systems in a completely native manner, allowing for robust, authentic challenge.^2, 7^

In this study, we stabilized the bRSV F glycoprotein trimer in its pre-F conformation by transplanting pre-F-stabilizing mutations previously designed for hRSV F^17, 18^ into bRSV F. Despite high-structural similarity and high-sequence identities of greater than 80% between hRSV and bRSV F glycoproteins, transplantation was not trivial. Out of seven bRSV strains evaluated for DS-Cav1 transplantation, only three expressed at levels high enough to permit characterization. Furthermore, the introduction of highly stable sc and DS2-disulfide mutations into bRSV F resulted in even lower expression yields, necessitating extensive screening of nearly 100 designs. Our results suggest that the transplantation of stabilizing pre-F mutations even for relatively closely related paramyxoviruses faces hurdles of antigenic stability and expression yield. Nevertheless, our design efforts for bRSV F resulted in a highly stable immunogen recognized by pre-F-specific RSV F antibodies that was immunogenic in mice and protective in calves.

Remarkably, the neutralization titers elicited by the DS2 immunogen in calves (geometric mean 21,849 at week 8) were >100 higher than those elicited by the post-F immunogen. This is consistent with neutralization titers elicited by hRSV F DS2 immunogens in mice which were also >100 higher than post-F elicited titers.^18^ In contrast, the overall immunogenicities of pre-F DS2 and post-F bRSV F immunogens as measured by ELISA were statistically comparable (Supplementary Fig. 3), suggesting that differences in elicited neutralization titers were due to differences in antibody neutralization potency, which likely reflects the availability and stability of neutralization-sensitive epitopes on the immunogens. The epitopes recognized by the potent neutralizing antibodies D25 and AM14 are present exclusively in the pre-F conformation^33^, whereas epitopes for less potent neutralizing antibodies such as Pz, (10–100 less potent),^17^ are present in both the pre-F and post-F conformations. The low neutralization titers induced by post-F bRSV F are not unprecedented as a recent study has reported similarly low values.^18^ Moreover, there is a significant correlation between pre-F stability and the ability to elicit neutralizing antibodies.^17, 18^ Thus, it is not surprising that DS-Cav1 versions of hRSV F and bRSV F, which are less thermostable than the DS2 versions, consistently elicit neutralization titers lower than DS2 pre-F immunogens^17, 18^ (Supplementary Table 7). Interestingly, two recent human clinical trials evaluating pre-F and post-F versions of recombinant hRSV F, respectively, revealed roughly comparable neutralization titers for similar time points and doses.^35, 36^ However, the post-F immunogen was presented as a nanoparticle, which may have enhanced its immunogenicity, whereas the pre-F immunogen was not in a nanoparticle format and its degree of pre-F stabilization was unclear. Finally, one caveat that makes it difficult to directly compare multiple immunogenicity studies in general is that differences in the neutralization assay, including detection method, target cell line, and the virus strain can affect the dynamic range of the assay results.

Upon challenge with the heterologous bRSV Snook strain, significant differences were observed between the DS2-immunized and post-F-immunized groups of calves. Vaccination with the DS2 immunogen protected four out of five calves against clinical signs of disease and pulmonary inflammation induced by bRSV infection and appeared to protect all immunized calves against bRSV replication in both the upper and lower respiratory tract. However, an increase in RR on days 5 and 6, together with the presence of macroscopic lung lesions, and a high proportion of neutrophils in the BAL of one of the calves vaccinated with the DS2 immunogen suggests a lack of protection in this animal from bRSV, and the failure to isolate bRSV from this animal could have been due to *in vitro* neutralization of virus infectivity. Vaccination with a post-F immunogen also appeared to reduce the severity of clinical signs of disease, the extent of macroscopic lung lesions, and replication of bRSV in the lung. However, protection against bRSV infection in calves vaccinated with post-F immunogen did not appear to be as great as that in animals vaccinated with the DS2 immunogen.

Intramuscular vaccination is unlikely to have induced a mucosal immune response which restricted virus infection at the portal of entry. Therefore, the failure to isolate bRSV from the nasopharynx of DS2-immunized calves may have been due to transudation of serum antibodies. However, the absence of a pulmonary inflammatory response in the majority of DS2-immunized calves indicated bRSV replication to be severely restricted in the lower respiratory tract.

Importantly, vaccination with the DS2 immunogen did not prime for enhanced respiratory disease (ERD) when calves were challenged, 4 weeks after vaccination. Furthermore, pulmonary eosinophilia, which has been associated with vaccine-ERD in infants, calves, and mice,^11, 37, 38^ was not seen in either group of vaccinated calves, even though bRSV was isolated from the BAL of all calves that had been vaccinated with post-F bRSV F. ERD in mice and cotton rats vaccinated with formalin-inactivated virus or purified F protein is associated with virus replication in the lungs, induction of a Th2-biased cytokine response and low affinity, poorly neutralizing antibodies.^38–40^ Although the T-cell response was not analyzed in the present study, the Montanide^TM^ ISA 71G adjuvant used was designed to increase Th1 responses in veterinary species.

There is still a great need for an effective commercial bRSV vaccine, as evidenced by the considerable annual economic and production losses to the cattle industry from bRSV.^3^ Furthermore, a recent meta-analysis of vaccination studies reported no significant difference in mortality and morbidity risk between unvaccinated calves and those vaccinated with modified live bRSV vaccines and mixed results for inactivated bRSV vaccines.^41^ The robust protection of calves from bRSV elicited by vaccination with a pre-F-stabilized DS2 immunogen provides proof-of-concept that such a structurally engineered immunogen can be effective against RSV infection in a native host. Moreover, the close evolutionary relationship between bRSV and hRSV suggests that a human DS2 immunogen could similarly protect humans from hRSV. Indeed, recently engineered hRSV DS2-stabilized pre-F immunogens have also been reported to elicit high-neutralization titers to hRSV in mice.^18^ Although there are theoretical concerns that subunit vaccines could prime for ERD in hRSV-naive infants, DS2 was not observed to induce ERD in any immunized calves and recombinant viral vectors expressing DS2 could be delivered at a mucosal surface where it would be expected to prime both CD4 and CD8 T cells, thus mitigating the potential for ERD, as well as inducing high levels of neutralizing antibodies. Vaccines consisting of post-F hRSV F protein are currently in development to boost immunity in the elderly,^42^ and to vaccinate pregnant women in order to boost protection against hRSV in young infants.^43^ However, the demonstration that pre-F-stabilized DS2 bRSV F induces levels of neutralizing antibodies in calves that are more than 100-fold greater than those induced by post-F immunogens indicates that significantly higher levels of protection may be achievable in humans through vaccination with DS2-like hRSV F immunogens. Together, our results support the further evaluation of vaccines based on DS2-stabilized forms of RSV F in both humans and cattle.

## Materials and methods

### Protein expression, purification

RSV F variants were expressed by transient transfection of Expi293F cells (Thermo Fisher Scientific, MA) using 293Fectin (Thermo Fisher Scientific, MA). Cell culture supernatants were harvested 5 days post transfection and centrifuged at 10,000×*g* to remove cell debris. The supernatants were sterile-filtered, and RSV F variants were purified by nickel (Roche) and Strep-Tactin (iba) affinity chromatography followed by SEC. The foldon domain was removed only when proteins were prepared for animal immunization. The C-terminal tags were removed from the variants by digestion with 2 U/ml restriction-grade thrombin (Novagen) overnight at 4 °C. The bRSV F glycoprotein with purification tags removed were then purified by a second round of SEC in PBS.

### Expression and purification of antibodies and antigen-binding fragments (Fabs)

Antibodies were expressed by transient co-transfection of Expi293F cells with both heavy- and light-chain plasmids using 293fectin. Cell supernatants were harvested after 4–5 days and passed over Protein A agarose (GE Healthcare, PA). Bound antibodies were washed with PBS and eluted with IgG elution buffer (Pierce, IL) into 1/10th volume of 1 M Tris-HCl pH 8.0. Fabs were generated by digesting the IgG with Lys-C or HRV3C protease,^17^ and the cleaved Fc region was removed by passing the mixture over Protein A agarose. Final purification of Fabs was performed by SEC.

### Antigenic screening of bRSV F immunogens

Initial assessment of all constructs were performed using a 96-well microplate format for high throughput expression followed by an ELISA-based antigenic evaluation as described previously.^17^ Briefly, 24 h prior to transfection HEK 293T cells (Thermo Fisher Scientific, MA) were seeded in each well of a 96-well microplate at a density of 2.5×10^5^ cells/ml in expression medium (high glucose Dulbecco's modified Eagle medium (DMEM) supplemented with 10% ultra-low IgG fetal bovine serum and 1×-non-essential amino acids), and incubated at 37 °C, 5% CO_2_ for 20 h. Plasmid DNA and TrueFect-Max (United BioSystems, MD) were mixed and added to the growing cells, and the 96-well plate incubated at 37 °C, 5% CO_2_. One day post transfection, enriched medium (high glucose DMEM plus 25% ultra-low IgG fetal bovine serum, 2× nonessential amino acids, 1× glutamine) was added to each well, and the 96-well plate was returned to the incubator for continuous culture. Five days post transfection supernatants with the expressed bRSV F variants were harvested and tested by ELISA for binding to D25, MPE8 and Mz antibodies using Ni^2+^-nitrilotriacetic acid (NTA) microplates.

### RSV F antigenic characterization

A fortéBio Octet Red384 instrument was used to measure binding kinetics of RSV F variants to antibodies that target the pre-F or post-F form (D25, AM14, MPE8, and Mz). All assays were performed with agitation set to 1000 rpm in PBS supplemented with 1% bovine serum albumin (BSA) to minimize nonspecific interactions. The final volume for all solutions was 50 μl/well. Assays were performed at 30 °C in tilted black 384-well plates (Geiger Bio-One). Ni-NTA sensor tips were used to capture relevant RSV F variants. Typical capture levels for each loading step were between 1.4 and 1.5 nm, and variability within a row of eight tips did not exceed 0.1 nm for each of these steps. Biosensor tips were equilibrated for 120 s in PBS + 1% BSA prior to loading bRSV F variants. Biosensor tips were then equilibrated for 120 s in PBS + 1% BSA prior to measuring association with Fabs in solution (0.007 μM to 1.0 μM) for 300 s; Fabs were then allowed to dissociate for 300–1200 s depending on the observed dissociation rate. Parallel correction to subtract systematic baseline drift was carried out by subtracting the measurements recorded for a loaded sensor incubated in PBS + 1% BSA. Data analysis and curve fitting were carried out using Octet software, version 9.0. Experimental data were fitted with the binding equations describing a 1:1 interaction. Global analysis of the data sets assuming reversible binding (full dissociation) were carried out using nonlinear least-squares fitting allowing a single set of binding parameters to be obtained simultaneously for all of the concentrations used in each experiment.

### Physical stability of RSV F variants

To assess the physical stability of the pre-F conformation of designed bRSV F glycoproteins under various stress conditions, we treated the proteins with a variety of pharmaceutically relevant stresses such as extreme pH, high temperature, low and high osmolarity, and repeated freeze/thaw cycles while at a concentration of 50 μg/ml. The physical stability of treated bRSV F variants was evaluated by the preservation of antigenic site Ø after treatment as assessed by binding of the site Ø-specific antibody D25. In pH treatments, the bRSV F glycoprotein solution was adjusted to pH 3.5 and pH 10 with appropriate buffers and incubated at room temperature for 60 min and subsequently neutralized to pH 7.5. Temperature treatments were carried out by incubating the bRSV F glycoprotein solutions at 50 °C and 70 °C for 60 min in a PCR cycler with heated lid. In osmolarity treatments, bRSV F glycoprotein solutions originally containing 150 mM NaCl were either diluted with 2.5 mM Tris buffer (pH 7.5) to an osmolarity of 10 mM NaCl or adjusted with 4.5 M MgCl_2 _to a final concentration of 3.0 M MgCl_2_. Protein solutions were incubated for 60 min at room temperature and then returned to 150 mM salt by adding 5.0 M NaCl or dilution with 2.5 mM Tris buffer, respectively, and concentrated to 50 μg/ml. The freeze/thaw treatment was carried out by repeatedly freezing bRSV F glycoprotein solutions in liquid nitrogen and thawing at 37 °C ten times in the presence of 10% glycerol. All bRSV F glycoproteins were diluted to 40 μg/ml with PBS + 1% BSA, and their ability to bind D25 Fab was measured with an Octet instrument using the protocol described above. The degree of physical stability is reported as the ratio of steady state D25-binding level before and after stress treatment.

### Negative stain EM

Samples were diluted to approximately 0.01 mg/ml, adsorbed to freshly glow-discharged carbon-coated grids, rinsed with several drops of buffer containing 10 mM HEPES, pH 7.0, and 150 mM KCl, and stained with 0.75% uranylformate. Images were recorded on an FEI T20 microscope with a 2k × 2k Eagle CCD camera at a pixel size of 2.2 Å. Reference-free 2D classification and averaging were performed with EMAN2^44^ and SPIDER^45^.

### Crystallization and X-ray data collection of pre-F-stabilized bRSV F proteins

Crystallization conditions were screened by vapor diffusion using a Mosquito crystallization robot (TTP labtech) that generated sitting drops at 20 °C by mixing 0.2 μl of bRSV immunogens with 0.2 μl of reservoir solution. Optimized crystals for data collection were grown by manually setting up hanging drops combining 0.5 µl protein with 0.5 µl of reservoir solution. ATue51908 DS-Cav1 crystals were grown in 12% (w/v) PEG 3350, and 0.1 M sodium acetate pH 5.5, and DS2-v1 (391-2 sc9 DS-Cav1 Q98C Q361C) crystals were grown in 0.9 M K/Na tartrate, 0.16 M Li_2_SO_4_, and 0.1 M CHES pH 9.5. Prior to data collection, ATue51908 DS-Cav1 crystals were transferred to 15% (v/v) 2R,3R-butanediol, 18% (w/v) PEG 3350, and 0.1M sodium acetate pH 5.5 and DS2-v1crystals were transferred to 15% (v/v) 2R,3R-butanediol, 1.3 M K/Na tartrate, 0.16 M Li_2_SO_4_, and 0.1 M CHES pH 9.5 followed by flash freezing in liquid nitrogen. X-ray diffraction data were collected at a wavelength of 1.00 Å at the SER-CAT beamline ID-22 (Advanced Photon Source, Argonne National Laboratory).

### Structure determination, refinement, and analysis of pre-F-stabilized bRSV F

Diffraction data were integrated and scaled with the HKL2000 suite,^46^ and a molecular replacement solutions for both structures were obtained by PHASER^47^ using the pre-F RSV F structure (PDB ID: 4MMS^17^) as a search model. Manual model building was carried out using COOT,^48^ with secondary structure elements built first. Refinement of individual coordinates, TLS parameters, and individual B-factors was performed in PHENIX.^49^ Final data collection and refinement statistics are presented in Supplementary Table 6. All structural images were created using PyMol (The PyMol Molecular Graphics System, version 1.1; Schrödinger, LLC).

### Mouse immunizations

All mouse experiments were reviewed and approved by the Animal Care and Use Committee of the Vaccine Research Center, NIAID, NIH, under animal protocol 13–454, and all animals were housed and cared for in accordance with local, state, federal, and institute policies in an American Association for Accreditation of Laboratory Animal Care (AAALAC)-accredited facility at the NIH. Mice were randomized into groups of ten and these groups were not blinded to the investigators. As in previous experiments,^17^ hybrid female mice that were the first filial offspring of a cross between BALB/cJ females (C) and C57BL/6J males (B6) (The Jackson Laboratory) known as CB6F1/J at ages 6 weeks to 12 weeks were intramuscularly injected with RSV F immunogens at week 0 and week 3. The frozen RSV F variant immunogen proteins were thawed on ice and mixed with fivefold w/w poly I:C (Invivogen) adjuvant (that is, 10 μg RSV F, 50 μg Poly I:C per animal per immunization), with injections taking place within 1 h of immunogen:adjuvant preparation. No adverse effect from immunization was observed. Blood was collected at least 3 days before immunization, and at week 2, week 5, and week 7 post initial immunization.

### bRSV neutralization assays

bRSV microneutralization assay was performed using BT cells (ATCC CRL1390) and 500–1000 TCID50 (50% tissue culture infectious doses) of bRSV, strain 375 (ATCC VR1339). Briefly, immune sera were serially diluted in quadruplicates prior to mixing with 500–1000 TCID50 of bRSV for 1 h at 37 °C in a humidified 5% CO_2_ atmosphere prior to addition to monolayers of BT cells seeded the day before at 8000 cells/well. Cells were then incubated for 7 days, fixed with 70% methanol, stained with 1% crystal violet and examined at the microscope for syncytia formation and cytopathic effect (CPE). Neutralizing titer was defined as the reciprocal of the highest sera dilution at which the infectivity of bRSV was completely neutralized in 50% of the wells. Infectivity was identified by the presence of CPE and syncytia on day 7, and the titer was calculated by the Reed-Muench method.

### ELISA-binding assays

A standard ELISA was used to determine binding of immune sera to bRSV pre- and post-bRSV F proteins. Briefly, ELISA plates were coated with antigens at 5 µg/ml, blocked with 1% BSA in PBS, incubated with serial dilutions of sera and washed. Bound mAbs were detected by incubation with AP-conjugated Goat Anti-Mouse adsorbed against human IgG (Southern Biotech) or goat anti-bovine IgG (Southern Biotech). Plates were then washed, substrate (4-Nitrophenyl phosphate disodium salt hexahydrate, Sigma) was added and plates were read at 405 nm. The relative titer of sera binding to respective coated antigens were determined by measuring the concentration of each serum required to achieve 50% binding relative to the maximum (ED_50_). The ED_50_ values were calculated by interpolation of binding curves fitted with a four-parameter nonlinear regression with a variable slope.

### Calf immunization

The calf experiment was performed under the regulations of the Home Office Scientific Procedures Act (1986) of the United Kingdom. The study had been reviewed and approved by the Animal and Plant Health Agency (APHA) Ethical Review Committee. Calve groups were not blinded to the investigators. Male calves were obtained from local farms and were removed from their mothers at birth to ensure that they did not receive any colostrum and transported to APHA at ~1 day of age. Calves were bled on arrival at APHA and were fed 250 ml of colostrum, 48 h after birth, in order produce calves with little or no maternally derived bRSV-specific serum antibodies. Sera obtained before and after colostrum intake was analyzed for bRSV-specific and prefusion bRSV F protein-specific antibodies by ELISA.^50^ All but two calves were free from bRSV-specific serum antibodies. Calves were allocated to three groups of five to give groups matched for calf age, and the two animals with maternally derived bRSV-specific antibodies were allocated to the control group. Calves were 3 to 6-weeks-old at the time of vaccination. The frozen bRSV F proteins, pre-F (DS2) and post-F (391-2 post-F) were thawed on ice and mixed with Montanide^TM^ ISA71 VG (Seppic, France) in a water in oil emulsion in a ratio of 70:30 adjuvant to aqueous phase. Calves were inoculated intramuscularly with 50 μg protein in a volume of 2 ml on two occasions 4 weeks apart. As controls, calves were inoculated with 2 ml PBS in ISA71 VG. Vaccinations took place within 3 h of immunogen:adjuvant preparation. Calves developed a transient fever 24 h after vaccination and no or only mild diffuse swelling at the injection sites. Calves were bled at defined time points for analysis of bRSV-specific serum antibody responses.

### Calf challenge virus

Virulent bRSV used to challenge calves consisted of BAL prepared from a gnotobiotic calf inoculated 6 days previously with the Snook strain of bRSV, which had been passaged on four previous occasions in gnotobiotic or specific pathogen free calves. The BAL was free from other viruses, mycoplasmas, and bacteria as assessed by inoculation of tissue culture cells, mycoplasmal or bacterial media. Virus titers were determined by plaque assay on fetal calf kidney cells.

### Calf challenge

Four weeks after the last vaccination, calves were challenged by intranasal and intratracheal administration of 10^4^ pfu of bRSV, Snook strain, in BAL.^50^ Following bRSV challenge, nasopharyngeal swabs were obtained daily to monitor bRSV excretion, and calves were examined daily for clinical signs of disease. The severity of disease was given a score as shown in Supplementary Table 12. The clinical scores are defined in Supplementary Table 13. Calves were euthanized 6 days after challenge to determine the extent of gross pneumonic consolidation and the extent of virus infection in the lower respiratory tract as described previously.^50^ Titers of virus in the trachea were determined by scraping the epithelium from a piece of trachea approximately 3 cm long into 2 ml of Hanks balanced salt solution (Sigma) containing 1% BSA (Sigma). The apical and cardiac lung lobes were clamped and the lungs lavaged with ~1 liter of PBS to obtain bronchalveolar lavage (BAL). Cytospin preparations of BAL cells were fixed and stained with Diff Quik (Thermo Fisher Scientific) and differential cell counts made using oil immersion microscopy. Samples of lung taken from three different apical lung lobes were homogenized to give a 20% w/v suspension. Lung tissue for histology was also taken from three different apical lobes and fixed in 10% neutral buffered formalin, paraffin wax embedded and sections were stained with hematoxylin and eosin.

### Statistical analysis

Statistical analyses were performed using two-tailed Mann–Whitney tests with GraphPad Prism 6.0 software (La Jolla, CA). Differences were considered statistically significant at *P* < 0.05.

## Electronic supplementary material

Supplementary Information
