# Supplementary material for: Protection of calves by a prefusion-stabilized bovine RSV F vaccine
Source: NPJ Vaccines. 2017 Mar 8;2:7. doi: 10.1038/s41541-017-0005-9 (PMC5627276; doi:10.1038/s41541-017-0005-9)
Supplement: Supplementary file 1 — Supplementary Information [file 41541_2017_5_MOESM1_ESM.pdf]

Supplementary Information

Protection of calves by a prefusion-stabilized bovine RSV F vaccine

Baoshan Zhang[1*], Lei Chen[1*], Chiara Silacci[2], Michelle Thom[3], Jeffrey C. Boyington[1], Aliaksandr Druz[1], M. Gordon Joyce[1], Efrain Guzman[3], Wing- Pui Kong[1], Yen-Ting Lai[1], Guillaume B. E. Stewart-Jones[1], Yaroslav Tsybovsky[4], Yongping Yang[1], Tongqing Zhou[1], Ulrich Baxa[4], John R. Mascola[1], Davide Corti[2,5], Antonio Lanzavecchia[2,6#], Geraldine Taylor[3#], Peter D. Kwong[1#]

[1]Vaccine Research Center, National Institute of Allergy and Infectious Diseases, National Institutes of Health, Bethesda, Maryland, United States of America
[2] Institute for Research in Biomedicine, Università della Svizzera italiana, 6500 Bellinzona, Switzerland
[3] The Pirbright Institute, Ash Road, Pirbright, Woking, Surrey GU24 0NF, United Kingdom
[4] Electron Microscopy Laboratory, Cancer Research Technology Program, Leidos Biomedical Research, Inc., Frederick National Laboratory for Cancer Research, Frederick, Maryland, United States of America
[5] Humabs BioMed SA, Via Mirasole 1, 6500 Bellinzona, Switzerland
[6] Institute for Microbiology, ETH Zurich, Wolfgang-Pauli-Strasse 10, 8093 Zurich, Switzerland

* Equal contribution
[#] Correspondence related to neutralization should be addressed to A Lanzavecchia (lanzavecchia@irb.usi.ch), related to calves to G Taylor (geraldine.taylor@pirbright.ac.uk), and related to structure-based vaccine design to PD Kwong (pdkwong@nih.gov).

Supplementary Table 1. Strain names and accession numbers for human and bovine RSV F proteins.
Supplementary Table 2. Expression screen for bDS-Cav1 RSV F in seven different strains.
Supplementary Table 3. Antigenic screening of bRSV F single chain variants.
Supplementary Table 4. Antigenic screening of bRSV F single chain immunogens with interprotomer disulfides.
Supplementary Table 5. Yields of bRSV single chain immunogens in liter-scale production.
Supplementary Table 6. Crystallographic data collection and refinement statistics.
Supplementary Table 7. bRSV neutralization IC_{50} titers measured from week 5 mouse sera.
Supplementary Table 8. Biographical data for immunized calves.
Supplementary Table 9. bRSV neutralization IC_{50} titers measured from calf sera.
Supplementary Table 10. Viral titers as a measure of bRSV replication in nasopharyngeal excretion.
Supplementary Table 11. Viral titers as a measure of bRSV replication in the respiratory tract of calves.
Supplementary Table 12. Clinical scores and signs of immunized calves.
Supplementary Table 13. Definition of clinical scores.
Supplementary Table 14. Effect of bRSV F vaccination on pulmonary pathology.
Supplementary Figure 1. Clustal Omega sequence alignment of hRSV F strain A2 with RSV F from nine bovine strains.
Supplementary Figure 2. Protein purification and electron microscopy analysis.
Supplementary Figure 3. Immunogenicity of engineered bovine RSV F pre-F trimers.
Supplementary Figure 4. Blocking of neutralizing antibody binding.
Supplementary Figure 5. Effect of vaccination on clinical signs of disease and neutrophils in BAL.
Supplementary Figure 6. Histology of lung sections from vaccinated calves.

Supplementary Table 1. Strain names and accession numbers for human and bovine RSV F proteins.

	Strain or isolate	GenBank accession number of RSV F protein
Human RSV	A/A2	AAB86664.1
	A/Long	ACO83302.1
	A/9830	AGL96787.1
	A/9846	AFP99057.1
	B/18537	BAA00240.1
	B/9717	AFP99062.1
	B/9320	AAR14266.1
Bovine RSV	ATue51908	NP_048055.1
	RB94	CAN90052.1
	A/375	ACL80037.1
	A51908	AAA42804.1
	Snook	CAA76980.1
	391-2 (also known as Copenhagen)	AAA42808.1
	RB94 F-11	BAA00798.1
	FS1	AAB28458.1
	ATCC 51908	AAL49399.1

Supplementary Table 2. Expression screen for bDS-Cav1 RSV F in seven different strains.

Name	Yield (mg/L)	Antibody Binding		
		D25	MPE8	Mz
RB94 DS-Cav1	0.76	+	+	+
ATue51908 DS-Cav1	2.98	+	+	+
391-2 DS-Cav1	3.66	+	+	+
A/375 DS-Cav1	0.12	ND	ND	ND
FS1 DS-Cav1	0.06	ND	ND	ND
ATCC 51908 DS-Cav1	0.05	ND	ND	ND
A51908 DS-Cav1	0.12	ND	ND	ND

+ Recognized by respective monoclonal antibody at a response level > 0.5 nm in an biolayer interferometry Octet binding analysis.

ND, not determined due to low yield.

Supplementary Table 3. Antigenic screening of bRSV F single chain immunogens.

Variant Number	bRSV F variants	D25	Mz	MPE8	MPE8+D25*
sc-v1	sc9-10_DS-CAV1_bRSV(RB94)_Fd_hp2_fp2_ig1	2.37	2.42	2.30	4.67
sc-v2	sc9-10_DS-CAV1_bRSV(RB94)_Fd_hp1_ig1	2.44	2.42	2.18	4.62
sc-v3	sc9-10_DS-CAV1_bRSV(RB94)_Fd_hp2_ig1	2.43	2.52	2.21	4.64
sc-v4	391-2-site 0 hRSV bovSurf DS-Cav1-BZGJ9 Long	2.30	2.27	2.19	4.49
sc-v5	sc9-10_DS-CAV1_bRSV(RB94)_Fd_hp2_fp1_ig1	2.17	2.18	2.25	4.42
sc-v6	RB94 site 0 hRSV DS-Cav1-BZGJ9 Long FdPL	2.16	2.26	2.20	4.36
sc-v7	RB94 site 0 hRSV bovSurf DS-Cav1-BZGJ9 Long FdPL	2.21	2.21	2.14	4.34
sc-v8	sc9-10_DS-CAV1_bRSV(RB94)_Fd_hp1_fp1_ig1	2.17	2.22	2.16	4.32
sc-v9	391-2-site 0 hRSV DSCav1-BZGJ9 Long	2.02	2.08	2.08	4.09
sc-v10	391-2-DS-Cav1-BZGJ9_gyc5	1.93	2.04	1.80	3.73
sc-v11	sc9-10_DS-CAV1_bRSV(RB94)_Fd_pm1_ig1	1.86	2.22	1.87	3.73
sc-v12	391-2-DS-Cav1-BZGJ9 Long K226L	1.92	1.94	1.78	3.70
sc-v13	sc9-10_DS-CAV1_bRSV(RB94)_Fd_hp1_fp2_ig1	1.88	1.80	1.81	3.68
sc-v14	RB94 site 0 hRSV bovSurf DS-Cav1 Long FdPL	1.70	1.86	1.84	3.54
sc-v15	sc9-10_DS-CAV1_bRSV(RB94)_Fd_pm1_fp1_ig1	1.67	1.81	1.73	3.40
sc-v16	391-2-DS-Cav1-BZGJ9_gyc1	1.40	2.12	1.55	2.95
sc-v17	391-2-DS-Cav1-BZGJ9 Long	1.30	1.55	1.45	2.75
sc-v18	391-2 hu/bov-DS-Cav1-BZGJ9 Long	1.39	1.70	1.32	2.72
sc-v19	391-2-DS-Cav1-IP-5	1.28	1.83	1.41	2.70
sc-v20	RB94 hu/bov DS-Cav1 Long FdPL	1.25	1.44	1.38	2.64
sc-v21	391-2_DS-Cav1-tzsc7	1.00	1.99	1.50	2.50
sc-v22	RB94 site 0 hRSV DS-Cav1 Long FdPL	1.02	1.75	1.39	2.42
sc-v23	391-2-DS-Cav1-BZGJ9 Long S190V	1.13	1.31	1.19	2.32
sc-v24	sc9-10_DS-CAV1_bRSV(RB94)_Fd_pm1_fp2_ig1	1.01	1.32	0.97	1.98
sc-v25	391-2_DS-Cav1-tzsc5	0.66	1.57	1.22	1.88
sc-v26	391-2wt-F_SC_11R	0.74	1.79	0.89	1.63
sc-v27	391-2-DS-Cav1-BZGJ9_gyc7	0.73	0.98	0.82	1.55
sc-v28	391-2wt_F-SC_12R core	0.71	0.82	0.65	1.36
sc-v29	KXint1	0.42	0.59	0.60	1.02
sc-v30	KXlinker1	0.40	0.83	0.54	0.94
sc-v31	RB94 DS-Cav1 Long FdPL	0.39	0.84	0.49	0.88
sc-v32	391-2-DS-Cav1-IP-4	0.33	0.93	0.54	0.87
sc-v33	391-2_DS-Cav1-tzsc3	0.38	0.78	0.43	0.82
sc-v34	sc9-10_DS-CAV1_bRSV(RB94)_Fd_fp2_ig1	0.30	0.75	0.46	0.76
sc-v35	KXlinker2	0.32	0.75	0.36	0.68
sc-v36	391-2-DSCav1- F-SC_7R	0.22	0.65	0.31	0.54
sc-v37	RB94_sc9-10 DS-CAV1 L373R	0.27	0.69	0.26	0.53
sc-v38	391-2_DS-Cav1-tzsc4	0.22	0.64	0.26	0.48
sc-v39	391-2-DS-Cav1-IP-2	0.20	0.56	0.22	0.41
sc-v40	RB94 DS-CAV1 N70K S104F K117T	0.17	0.27	0.20	0.37
sc-v41	sc9-10_DS-CAV1_bRSV(RB94)_Fd_(hRSV-leader)_ig1	0.20	0.54	0.11	0.30
sc-v42	sc9-10_DS-CAV1_bRSV(RB94)_Fd_fp1_ig1	0.19	0.54	0.10	0.29
sc-v43	391-2-DS-Cav1-BZGJ9_gyc3	0.16	0.60	0.12	0.29
sc-v44	391-2-DS-Cav1-IP-7	0.20	0.55	0.08	0.28
sc-v45	391-2-DS-Cav1-BZGJ9	0.18	0.50	0.09	0.28
sc-v46	391-2-DS-Cav1-BZGJ9_gyc2	0.14	1.58	0.12	0.26
sc-v47	391-2-DS-Cav1-SC-IP-1	0.10	1.10	0.16	0.25
sc-v48	KXds2	0.11	0.36	0.14	0.25
sc-v49	391-2_DS-Cav1-tzsc4	0.14	0.50	0.10	0.23
sc-v50	391-2_DS-Cav1-tzsc9	0.13	1.59	0.10	0.23
sc-v51	391-2-DS-Cav1-BZGJ9_gyc4	0.14	0.49	0.09	0.23
sc-v52	391-2-DS-Cav1- F-SC_11R	0.16	0.54	0.07	0.23
sc-v53	391-2-DS-Cav1-BZGJ9_gyc6	0.13	1.05	0.09	0.22
sc-v54	KXsb2	0.13	0.48	0.08	0.21
	media control	0.13	0.55	0.07	0.20
sc-v55	391-2_DS-Cav1-tzsc2	0.09	0.37	0.11	0.20
sc-v56	391-2_DS-Cav1-tzsc6	0.14	0.53	0.06	0.20
sc-v57	391-2-DS-Cav1-BZGJ9deleteFS	0.12	0.15	0.07	0.19
sc-v58	391-2-DS-Cav1-BZGJ9_gyc10	0.14	0.46	0.05	0.19

* Immunogens are ranked according to combined D25 and MPE8 ELISA binding values.

Supplementary Table 4. Antigenic screening of bRSV F single chain immunogens with interprotomer disulfides.

Variant Number	bRSV F variants	D25	Mz	MPE8	MPE8+ D25*
DS2-v1	391-2 sc9 DS-Cav1 Q98C Q361C	0.60	0.76	0.67	1.27
DS2-v2	391-2 sc9 DS-Cav1 Q98C S362C	0.43	0.75	0.48	0.91
DS2-v3	RB94 sc9 DS-CAV1 N183GC N428C	0.37	1.09	0.52	0.89
DS2-v4	391-2 DS-Cav1 sc9deleteFS Q98C S362C	0.36	0.59	0.45	0.81
DS2-v5	RB94 sc9 DS-CAV1 A149C Y458C	0.32	1.85	0.42	0.74
DS2-v6	391-2 DS-Cav1 sc9 E100C S362C	0.27	0.60	0.13	0.40
DS2-v7	RB94 hu/bov DS-CAV1 A149C Y458C Long	0.27	0.79	0.29	0.56
DS2-v8	391-2 DS-Cav1 sc9deleteFS Q98C Q361C	0.27	0.90	0.43	0.70
DS2-v9	RB94 sc9 DS-CAV1 N183GC N428C L373R Long	0.26	0.49	0.34	0.60
DS2-v10	391-2 DS-Cav1 sc9 N99C S362C	0.25	0.39	0.31	0.56
DS2-v11	391-2 DS-Cav1 sc9 N99C Q361C	0.22	1.68	0.20	0.42
DS2-v12	RB94 sc9 DS-CAV1 N183GC N428C L373R Long K226L	0.21	0.63	0.29	0.50
DS2-v13	RB94 sc9 DS-CAV1 N183GC N428C L373R Long S190V	0.21	0.62	0.16	0.36
DS2-v14	391-2 DS-Cav1 sc9 I28C G464C	0.20	0.57	0.20	0.41
DS2-v15	391-2 DS-Cav1 sc9deleteFS N99C Q361C	0.20	0.61	0.23	0.43
DS2-v16	391-2 DS-Cav1 sc9deleteFS N99C S362C	0.20	0.61	0.20	0.40
DS2-v17	RB94 sc9 DS-CAV1 N183GC N428C L373R	0.19	1.04	0.32	0.51
DS2-v18	RB94 dscav1T369C T455C Long S190V	0.18	0.51	0.37	0.55
DS2-v19	391-2 DS-Cav1-sc9 E100C Q361C	0.18	1.63	0.17	0.35
DS2-v20	RB94 sc9 DS-CAV1 A149C Y458C L373R	0.18	0.56	0.15	0.33
DS2-v21	391-2 DS-Cav1 sc9deleteFS E100C S362C	0.16	0.50	0.08	0.24
DS2-v22	RB94 sc9 DS-CAV1 A149C Y458C L373R Long	0.16	0.51	0.07	0.23
DS2-v23	391-2 DS-Cav1 sc9 S398C-S485C	0.14	0.49	0.08	0.23
DS2-v24	RB94 sc9 DS-CAV1 A149C Y458C L373R Long K226L	0.14	0.49	0.09	0.23
DS2-v25	391-2 DS-Cav1 S398C S485C	0.14	0.55	0.08	0.22
DS2-v26	391-2 DS-Cav1 I28C G464C	0.14	1.21	0.08	0.22
DS2-v27	391-2 DS-Cav1 sc9 I395C A490C	0.14	0.89	0.09	0.23
DS2-v28	391-2 DS-Cav1 sc9 S485C A490C	0.14	0.59	0.06	0.19
DS2-v29	391-2 DS-Cav1 sc9deleteFS 100C 361C	0.13	0.48	0.06	0.19
DS2-v30	RB94 sc9 DS-CAV1 A149C Y458C L373R Long S190V	0.13	0.54	0.05	0.18
DS2-v31	391-2 DS-Cav1 sc9 T449C V459C	0.13	0.49	0.10	0.23
DS2-v32	RB94 DS-CAV1 T369C T455C Long	0.13	0.45	0.07	0.20

* Immunogens are ranked according to combined D25 and MPE8 ELISA binding values.

Supplementary Table 5. Yields of bRSV single chain immunogens in liter-scale production.

	**Variant Number**	**bRSV F variant**	**Yield (mg/L)**
Single chain with interprotomer disulfide bond alterations	DS2-v1	391-2 sc9 DS-Cav1 Q98C Q361C	2.80
	DS2-v3	RB94 sc9 DS-CAV1 N183GC N428C	0.03
	DS2-v5	RB94 sc9 DS-Cav1 A149C Y458C	0.06
	DS2-v33	391-2 sc9-10 DS-Cav1 Q98C Q361C	2.44
	DS2-v34	391-2 sc9 DS-Cav1 A149C Y458C	0.05
	DS2-v35	ATue51908 sc9-10 DS-Cav1 A149C Y458C	0.24
	DS2-v36	ATue51908 sc9-10 DS-Cav1 N183GC N428C	0.03
Single chain	sc-v1	sc9-10_DS-CAV1_bRSV(RB94)_Fd_hp2_fp2_ig1	21.50
	sc-v4	391-2-site 0 hRSV bovSurf DS-Cav1-BZGJ9 Long	18.60

Supplementary Table 6. Crystallographic data collection and refinement statistics.

	ATue51980 DS-Cav1	DS2-v1
**PDB ID**	5TDG	5TDL
**Data collection**		
Space group	$P12_11$	$P4_132$
Cell constants		
a, b, *c* (Å)	73.1, 127.0, 92.4	172.1, 172.1, 172.1
α, β , γ (°)	90, 93.9, 90	90, 90, 90
Resolution (Å)	43.3-2.65 (2.74-2.65)	47.7-3.50 (3.59-3.50)
R_{merge} (%)	10.8 (76.1)	13.3 (105.7)
I / σ(I)	16.5 (2.1)	9.6 (2.0)
Completeness (%)	97.0 (98.5)	99.5 (100.0)
Redundancy	3.4 (2.9)	10.4 (11.1)
Wilson B	66.2	158.9
**Refinement**		
Resolution (Å)	43.3-2.65	40.6-3.50
No. reflections	47,383	11,449
R_{work} / R_{free} (%)	18.5/23.9	25.6/27.2
No. atoms		
Protein	11,204	3,484
Carbohydrate	42	14
Water	30	0
B-factors		
Protein	81.2	162.0
Carbohydrate	133.5	188.3
Water	73.0	NA
R.m.s. deviations		
Bond lengths (Å)	0.003	0.002
Bond angles (°)	0.579	0.490

A single crystal was used for each structure. Values in parentheses are for the highest resolution shell. NA, non-applicable.

Supplementary Table 7. bRSV neutralization EC_{50} titers measured from week 5 mouse sera.

Immunization group	DS2-v1	DS2-v33	391-2 DS-Cav1	391-2 post-F	ATue51908 DS-Cav1	DS2-v35	ATue51908 post-F	RB94 DS-Cav1	RB94 post-F
	1909	17183	1909	100	4243	5728	178	4243	141
	57276	17183	6815	100	12728	5728	212	11339	141
	5728	6815	1909	100	12728	5728	212	27000	141
	6815	5728	2272	100	27000	5728	150	4243	141
	17183	68148	68148	100	4243	17183	150	12728	1273
	14453	2272	4818	100	12728	14453	178	11339	100
	5728	25747	4818	100	38184	14453	212	3000	141
	17183	17183	20444	100	5072	14453	212	4243	100
	14453	2272	5728	100	4243	17183	636	11339	100
	25747	1909	22716	100	4243	5728	178	12728	141
**Geometric mean**	**11453**	**8984**	**6880**	**100**	**9002**	**9419**	**210**	**8250**	**158**

Undetectable levels of neutralization were assigned a value of 100.

Supplementary Table 8. Biographical data for immunized calves.

Immunization group	Calf no.	DoB*	Sex	Breed†	Age at study onset (days)	Age at study onset (weeks)
DS2-v1	603941	9/23/15	M	HF	42	6
	103943	9/28/15	M	HF	37	5
	303567	2/29/15	M	HF	36	5
	703571	10/8/15	M	HF	27	4
	303574	10/13/15	M	NRX	22	3
Post-F 391-2 post-F	703942	9/24/15	M	HF	41	6
	203566	9/27/15	M	HF	38	6
	503569	9/30/15	M	AA	35	5
	403946	10/9/15	M	AAX	26	4
	103572	10/13/15	M	HF	22	3
Placebo PBS	703564	9/24/15	M	HF	41	6
	103565	9/26/15	M	HF	39	6
	403568	9/29/15	M	HF	36	5
	603570	10/2/15	M	HF	33	5
	203573‡	10/13/15	M	HF	22	3

* DoB, date of birth.

† Breeds: HF, Holstein Friesians; NRX, Norwegian Red Cross; AA, Aberdeen Angus; AAX, Aberdeen Angus Cross.

‡ Calf 203573 was lost at 15 days post prime immunization due to a joint infection.

Supplementary Table 9. bRSV neutralization EC_{50} titers measured from calf sera.

Immunization	Calf no.	Week post immunization				
		0	2	4	6	8
DS2-v1	603941	100	357	1273	28905	11455
	103943	100	357	3212	96351	13630
	303567	100	141	424	38184	17165
	703571	100	424	1273	136296	136296
	303574	100	119	424	38184	13630
	Geometric Mean	100	246	987	56055	21849
Post-F 391-2 post-F	703942	100	100	100	424	213
	203566	100	100	100	100	119
	503569	100	100	100	100	141
	403946	100	100	100	168	424
	103572	100	100	100	168	100
	Geometric Mean	100	100	100	164	172
Placebo PBS	703564	100	100	100	100	100
	103565	141	141	100	100	100
	403568	424	505	424	505	141
	603570	100	100	100	100	100
	Geometric Mean	156	163	144	150	109

Undetectable levels of neutralization were assigned a value of 100.

Supplementary Table 10. Viral titers as a measure of bRSV replication in nasopharyngeal secretions.

Immunization	Calf no.	Viral titers (log_{10} pfu/ml)*						
		Days post inoculation						
		0	1	2	3	4	5	6
DS2-v1	603941	0.6	0.6	0.6	0.6	0.6	0.6	0.6
	103943	0.6	0.6	0.6	0.6	0.6	0.6	0.6
	303567	0.6	0.6	0.6	0.6	0.6	0.6	0.6
	703571	0.6	0.6	0.6	0.6	0.6	0.6	0.6
	303574	0.6	0.6	0.6	0.6	0.6	0.6	0.6
	**Mean**	**0.6**	**0.6**	**0.6**	**0.6**	**0.6**	**0.6**	**0.6**
	**sd**	**0.0**	**0.0**	**0.0**	**0.0**	**0.0**	**0.0**	**0.0**
Post-F	703942	0.6	0.6	0.6	0.6	0.6	1.0	0.6
391-2 post-F	203566	0.6	0.6	0.6	0.6	0.6	0.6	0.6
	503569	0.6	0.6	0.6	0.6	3.0	0.6	0.7
	403946	0.6	0.6	0.6	0.6	2.7	2.5	2.5
	103572	0.6	0.6	0.6	0.6	2.1	3.2	2.7
	**Mean**	**0.6**	**0.6**	**0.6**	**0.6**	**1.8**	**1.6**	**1.4**
	**sd**	**0.0**	**0.0**	**0.0**	**0.0**	**1.1**	**1.2**	**1.1**
Placebo	703564	0.6	0.6	0.6	0.6	0.6	2.6	0.6
PBS	103565	0.6	0.6	0.6	1.5	0.6	2.9	1.8
	403568	0.6	0.6	0.6	0.6	0.6	0.6	1.3
	603570	0.6	0.6	0.6	0.6	1.2	0.6	0.6
	**Mean**	**0.6**	**0.6**	**0.6**	**0.8**	**0.7**	**1.7**	**1.1**
	**sd**	**0.0**	**0.0**	**0.0**	**0.5**	**0.3**	**1.2**	**0.6**

Mean

	0	1	2	3	4	5	6
Pre-F	0.6	0.6	0.6	0.6	0.6	0.6	0.6
Post-F	0.6	0.6	0.6	0.6	1.8	1.6	1.4
Placebo	0.6	0.6	0.6	0.8	0.7	1.7	1.1

* Undetectable levels of virus were assigned a value of 0.6 .

Supplementary Table 11. Viral titers as a measure of bRSV replication in the respiratory tract of calves.

Immunization	Calf no.	Viral titers (Log_{10} pfu/ml or g)*				
		Respiratory tract samples†				
		TSc	LWC	RA	RC	LC
DS2-v1	603941	0.6	0.6	0.6	0.6	0.6
	103943	0.6	0.6	0.6	0.6	0.6
	303567	0.6	0.6	0.6	0.6	0.6
	703571	0.6	0.6	0.6	0.6	0.6
	303574	0.6	0.6	0.6	0.6	0.6
	**Mean**	**0.6**	**0.6**	**0.6**	**0.6**	**0.6**
	**sd**	**0.0**	**0.0**	**0.0**	**0.0**	**0.0**
Post-F	703942	0.6	1.7	0.6	0.6	0.6
391-2 post-F	203566	1.2	1.2	0.6	1.5	0.6
	503569	3.2	3.1	0.6	0.6	0.6
	403946	0.6	3.0	0.6	0.6	0.6
	103572	2.8	2.0	0.6	0.6	0.6
	**Mean**	**1.7**	**2.2**	**0.6**	**0.8**	**0.6**
	**sd**	**1.2**	**0.8**	**0.0**	**0.4**	**0.0**
Placebo	703564	2.5	1.3	2.5	1.3	2.0
PBS	103565	2.8	2.9	2.7	2.9	2.3
	403568	3.2	3.7	3.6	2.4	0.6
	603570	0.6	0.6	2.0	2.1	2.1
	**Mean**	**2.3**	**2.1**	**2.7**	**2.1**	**1.8**
	**sd**	**1.2**	**1.4**	**0.7**	**0.7**	**0.8**

Mean	TSc	LWC	RA	RC	LC
Pre-F	0.6	0.6	0.6	0.6	0.6
Post-F	1.7	2.2	0.6	0.8	0.6
Placebo	2.3	2.1	2.7	2.2	1.8

* Undetectable levels of virus were assigned a value of 0.6 .

† TrSc, tracheal epithelium; LWC, lung wash cells; RA, right apical lobe of the lung; RC, right cardiac lobe of the lung; LC, left cardiac lobe of the lung.

Supplementary Table 12. Clinical scores and signs of immunized calves.

**Clinical scores**					**Days post inoculation**				
		**-1**	**0**	**1**	**2**	**3**	**4**	**5**	6
**DS2-v1**	603941	0	0	1	0	0	1	3	2
	103943	0	0	0	1	0	0	0	0
	303567	0	0	0	1	0	0	0	1
	703571	0	0	9	0	0	1	0	1
	303574	0	0	0	0	0	0	1	1
	**Mean**	**0**	**0**	**2**	**0.4**	**0**	**0.4**	**0.8**	**1**
	**sd**	**0**	**0**	**4**	**0.5**	**0**	**0.5**	**1**	**0.7**
**Post-F 391-2 post-F**	703942	0	0	0	1	0	0	0	0
	203566	1	2	1	3	1	1	1	1
	503569	1	0	1	5	3	2	3	3
	403946	0	0	0	1	0	0	1	2
	103572	0	1	1	0	0	0	1	0
	**Mean**	**0.4**	**0.6**	**0.6**	**2**	**0.8**	**0.6**	**1**	**1**
	**sd**	**0.5**	**0.9**	**0.5**	**2**	**1**	**0.9**	**1**	**1**
**Placebo PBS**	703564	0	1	0	5	0	1	0	1
	103565	0	0	0	0	0	0	0	3
	403568	0	0	0	1	0	0	3	5
	603570	0	0	0	0	0	0	3	5
	**Mean**	**0**	**0.3**	**0**	**2**	**0**	**0.3**	**2**	**4**
	**sd**	**0**	**0.5**	**0**	**2**	**0**	**0.5**	**2**	**2**

**Respiratory rate**									
		**-1**	**0**	**1**	**2**	**3**	**4**	**5**	6
**DS2-v1**	603941	28	32	36	24	32	42	38	38
	103943	20	24	28	40	28	32	26	30
	303567	24	28	32	38	28	32	26	36
	703571	32	32	40	32	32	36	30	38
	303574	32	28	32	34	26	28	40	42
	**Mean**	**27.2**	**28.8**	**33.6**	**33.6**	**29.2**	**34.0**	**32.0**	**36.8**
	**sd**	**5.2**	**3.3**	**4.6**	**6.2**	**2.7**	**5.3**	**6.6**	**4.4**
**Post-F 391-2 post-F**	703942	28	24	32	40	28	28	34	32
	203566	40	40	44	50	38	40	38	42
	503569	40	32	40	48	40	36	38	32
	403946	28	24	28	40	26	34	36	40
	103572	32	36	36	30	30	28	36	32
	**Mean**	**33.6**	**31.2**	**36**	**41.6**	**32.4**	**33.2**	**36.4**	**35.6**
	**sd**	**6.1**	**7.2**	**6.3**	**7.9**	**6.2**	**5.2**	**1.7**	**5.0**
**Placebo PBS**	703564	28	24	28	42	30	36	28	40
	103565	24	28	32	32	30	30	30	40
	403568	28	24	32	32	28	30	40	50
	603570	32	28	32	28	26	32	36	52
	**Mean**	**28.0**	**26.0**	**31.0**	**33.5**	**28.5**	**32.0**	**33.5**	**45.5**
	**sd**	**3.3**	**2.3**	**2.0**	**6.0**	**1.9**	**2.8**	**5.5**	**6.4**

**Temperatures**									
		**-1**	**0**	**1**	**2**	**3**	**4**	**5**	6
**DS2-v1**	603941	37.9	38.6	38.5	38.4	37.8	38.3	38.3	37.6
	103943	37.9	37.4	38.3	38.2	38.3	38.3	38.4	37.6
	303567	37.3	38.6	37.9	38.7	38.2	38.0	38.0	37.6
	703571	38.0	38.6	40.1	37.9	38.7	38.2	38.1	38.2
	303574	38.0	37.8	38.2	38.0	38.0	38.2	38.4	39.3
	**Mean**	**37.8**	**38.2**	**38.6**	**38.2**	**38.2**	**38.2**	**38.2**	**38.1**
	**sd**	**0.3**	**0.6**	**0.9**	**0.3**	**0.3**	**0.1**	**0.2**	**0.7**
**Post-F 391-2 post-F**	703942	38.6	37.9	38.1	38.4	38.2	38.6	38.4	37.5
	203566	38.5	38.6	38.5	38.4	38.7	38.0	38.6	38.7
	503569	38.4	38.4	38.7	38.2	39.0	38.5	38.6	38.9
	403946	38.1	38.1	38.0	37.6	38.6	38.8	38.5	38.4
	103572	38.1	38.4	38.3	38	37.9	38.1	38.2	38.4
	**Mean**	**38.3**	**38.3**	**38.3**	**38.1**	**38.5**	**38.4**	**38.5**	**38.4**
	**sd**	**0.2**	**0.3**	**0.3**	**0.3**	**0.4**	**0.3**	**0.2**	**0.5**
**Placebo PBS**	703564	38.0	38.4	38.2	38.4	38.2	38.8	38.8	39.1
	103565	38.1	38.2	37.9	38.4	37.6	37.8	38.6	39.5
	403568	38.4	38.5	38.5	38.3	38.1	38.5	38.8	39.5
	603570	37.9	38.3	37.9	38.4	38.5	38.5	38.3	39.4
	**Mean**	**38.1**	**38.4**	**38.1**	**38.4**	**38.1**	**38.4**	**38.6**	**39.4**
	**sd**	**0.2**	**0.1**	**0.3**	**0.1**	**0.4**	**0.4**	**0.2**	**0.2**

Supplementary Table 13. Definition of clinical scores.

	Score					
**Clinical signs**	0	1	2	3	5	10
Temperature	For each 0.1°C > 39.3°C add a score of 1					
Nasal signs	None	Slight discharge	Moderate discharge	Copious discharge	NA	NA
Ocular signs	None	Slight discharge	Moderate discharge	Copious discharge	NA	NA
Cough	None	Occurs during handling	Spontaneous, infrequent	NA	NA	Persistent loud cough
Respiratory rate (RR)	<35 breaths/min	35-45 breaths/min	NA	45-55 breaths/min	55-65 breaths/min	>75 breaths/min
Dyspnoea	Normal	Slight	Moderate	NA	NA	Severe (stretched neck & open mouth breathing)
Feeding	Normal	NA	NA	NA	NA	Reluctant to feed
Behavior	Normal	Slight apathy (ears back, avoids handling)	Moderate apathy (head down, reluctant to move)	NA	NA	Sever apathy (unable to move, lying down)

NA, Not applicable.

Supplementary Table 14. Effect of bRSV F vaccination on pulmonary pathology.

Immunization	Calf no.	x10^6 cells/ml	%PMN*	No. bacterial colonies in 100 µl of BAL	%Lung lesions
**DS2-v1**	603941	2.1	21.9	2	0
	103943	1.3	3.8	TNTC†	0
	303567	2.2	5.6	0	0
	703571	1.8	2.0	33	0.3
	303574	2.0	65.2	0	16
	**Mean**	**1.9**	**19.7**		
	**sd**	**0.4**	**26.7**		
**Post-F 391-2 post-F**	703942	2.5	44.3	0	0
	203566	2.1	66.1	0	4
	503569	4.9	60.9	0	7
	403946	1.3	20.0	0	1
	103572	8.8	70.1	TNTC	6
	**Mean**	**3.9**	**52.3**		
	**sd**	**3.0**	**20.1**		
**Placebo PBS**	703564	26.0	66.8	0	11
	103565	8.7	80.7	0	21
	403568	8.1	69.6	0	19
	603570	11.0	77.7	0	33
	**Mean**	**13.5**	**73.7**		
	**sd**	**8.5**	**6.6**		

* %PMN, percentage polymorph nuclear neutrophils in BAL.
† TNTC, Too numerous to count.

```
hRSV A2 gb|AAB86664.1       -----MELLILKANAITTILTAVTFCFASGQNITEEFYQSTCSAVSKGYLSALRTGWYTS
bRSV gb|AAA42804.1          MATTTMRMII-SIILISTYVP----HITLCQNITEEFYQSTCSAVSRGYLSALRTGWYTS
bRSV gb|ACL80037.1          -----MRMII-SIILISTYVP----HITLCQNITEEFYQSTCSAVSRGYLSALRTGWYTS
bRSV emb|CAN90052.1         MATTTMRMII-SIIIIFIYVQ----HITLCQNITEEFYQSTCSAVSRGYLSALRTGWYTS
bRSV dbj|BAA00798.1         MATTAMRMII-SIIFISTYVT----HITLCQNITEEFYQSTCSAVSRGYLSALRTGWYTS
bRSV gb|AAB28458.1          MGTTAMRMVI-SIIFISTYVT----HITLCQNITEEFYQSTCSAVSRGYLSALRTGWYTS
bRSV gb|AAA42808.1          MAATAMRMII-SIIFISTYMT----HITLCQNITEEFYQSTCSAVSRGYLSALRTGWYTS
bRSV ref|NP_048055.1        MATTAMRMII-SIIFISTYVT----HITLCQNITEEFYQSTCSAVSRGYLSALRTGWYTS
bRSV gb|AAL49399.1          MATTAMRMII-SIIFISTYVT----HITLCQNITEEFYQSTCSAVSRGYLSALRTGWYTS
bRSV emb|CAA76980.1         MATTAMTMII-SIIFISTYVT----HITLCQNITEEFYQSTCSAVSRGYLSALRTGWYTS
                                 * ::* .   *   :      ::  ****************:*************

hRSV A2 gb|AAB86664.1       VITIELSNIKENKCNGTDAKVKLIKQELDKYKNAVTELQLLMQSTPATNNRARRELPRFM
bRSV gb|AAA42804.1          VVTIELSKIQKNVCNGTDSKVKLIKQELERYNNAVAELQSLMQNEPTSSSRAKRGIPESI
bRSV gb|ACL80037.1          VVTIELSKIQKNVCNGTDSKVKLIKQELERYNNAVVELQSLMQNEPTSSSRAKRGIPESI
bRSV emb|CAN90052.1         VVTIELSKIQKNVCNSTDSNVKLIKQELERYNNAVVELQSLMQNEPASSSRAKRGIPELI
bRSV dbj|BAA00798.1         VVTIELSKIQKNVCNSTDSNVKLIKQELERYNNAVVELQSLMQNEPASSSRAKRGIPELI
bRSV gb|AAB28458.1          VVTIELSKIQKNVCKSTDSKVKLIKQELERYNNAVIELQSLMQNEPASFSRAKRGIPELI
bRSV gb|AAA42808.1          VVTIELSKIQKNVCKSTDSKVKLIKQELERYNNAVIELQSLMQNEPASFSRAKRGIPELI
bRSV ref|NP_048055.1        VVTIELSKIQKNVCKSTDSKVKLIKQELERYNNAVVELQSLMQNEPASFSRAKRGIPELI
bRSV gb|AAL49399.1          VVTIELSKIQKNVCNSTDSKVKLIKQELERYNNAVVELQSLMQNEPASFSRAKRGIPELI
bRSV emb|CAA76980.1         VVTIELSKIQKNVCKSTDSKVKLIKQELERYNNAVVELQSLMQNEPASFSRAKRSIPELI
                            *:*****:*::* *:.**::********::*:*** *** ***. *:: .**:* :*. :

hRSV A2 gb|AAB86664.1       NYTLNNAKKTNVTLSKKRKRRFLGFLLGVGSAIASGVAVSKVLHLEGEVNKIKSALLSTN
bRSV gb|AAA42804.1          HYTRNSTKKFYGLMGKKRKRRFLGFLLGIGSAIASGVAVSKVLHLEGEVNKIKNALLSTN
bRSV gb|ACL80037.1          HYTRNSTKKFYGLMGKKRKRRFLGFLLGIGSAIASGVAVSKVLHLEGEVNKIKNALLSTN
bRSV emb|CAN90052.1         HYKRNSTKKFYGLMGKKRKRRFLGFLLGIGSAIASGVAVSKVLHLEGEVNKIKNALLSTN
bRSV dbj|BAA00798.1         HYKRNSTKKFYGLMGKKRKRRFLGFLLGIGSAIASGVAVSKVLHLEGEVNKIKNALLSTN
bRSV gb|AAB28458.1          HYPRNSTKRFYGLMGKKRKRRFLGFLLGIGSAIASGVAVSKVLHLEGEVNKIKNALLSTN
bRSV gb|AAA42808.1          HYTRNSTKRFYGLMGKKRKRRFLGFLLGIGSAIASGVAVSKVLHLEGEVNKIKNALLSTN
bRSV ref|NP_048055.1        HYTRNSTKKFYGLMGKKRKRRFLGFLLGIGSAVASGVAVSKVLHLEGEVNKIKNALLSTN
bRSV gb|AAL49399.1          HYTRNSTKKFYGLMGKKRKRRFLGFLLGIGSAIASGVAVSKVLHLEGEVNKIKNALLSTN
bRSV emb|CAA76980.1         HYTRNSTKKFYGLMGKKRKRRFLGFLLGIGSAIASGVAVSKVLHLEGEVNKIKNALLSTN
                            .*  *.:*:    :.*************:***:********************.******

hRSV A2 gb|AAB86664.1       KAVVSLSNGVSVLTSKVLDLKNYIDKQLLPIVNKQSCSISNIATVIEFQQKNNRLLEITR
bRSV gb|AAA42804.1          KAVVSLSNGVSVLTSKVLDLKNYIDKELLPKVNNHDCRISNIATVIEFQQKNNRLLEIAR
bRSV gb|ACL80037.1          KAVVSLSNGVSVLTSKVLDLKNYIDKKLLPKVNNHDCRISNIETVIEFQQKNNRLLEIAR
bRSV emb|CAN90052.1         KAVVSLSNGVSVLTSKVLDLKNYIDKELLPKVNNHDCQISNIATVIEFQQKNNRLLEIAR
bRSV dbj|BAA00798.1         KAVVSLSNGVSVLTSKVLDLKNYIDKELLPKVNNHDCKISNIATVIEFQQKNNRLLEIAR
bRSV gb|AAB28458.1          KAVVSLSNGVSVLTSKVLDLKNYIDKELLPKVNNHDCRISNIGTVIEFQQKNNRLLEIAR
bRSV gb|AAA42808.1          KAVVSLSNGVSVLTSKVLDLKNYIDKELLPKVNNHDCRISNIETVIEFQQKNNRLLEIAR
bRSV ref|NP_048055.1        KAVVSLSNGVSVLTSKVLDLKNYIDKELLPQVNNHDCRISNIETVIEFQQKNNRLLEIAR
bRSV gb|AAL49399.1          KAVVSLSNGVSVLTSKVLDLKNYIDKELLPKVNNHDCRISKIETVIEFQQKNNRLLEIAR
bRSV emb|CAA76980.1         KAVVSLSNGVSVLTSKVLDLKNYIDKELLPKVNNHDCRISNIATVIEFQQKNNRLLEIAR
                            **************************:*** **::.* **:* ***************:*

hRSV A2 gb|AAB86664.1       EFSVNAGVTTPVSTYMLTNSELLSLINDMPITNDQKKLMSNNVQIVRQQSYSIMSIIKEE
bRSV gb|AAA42804.1          EFSVNAGITTPLSTYMLTNSELLSIINDMPITNDQKKLMS-VCQIVRQQSYSIMSVLR-E
bRSV gb|ACL80037.1          EFSVNAGITTPLSTYMLTNSELLSLINDMPITNDQKKLMSSNVQIVRQQSYSIMSVVKEE
bRSV emb|CAN90052.1         EFSVNAGITTPLSTYMLTNSELLSLINDMPITNDQKKLMSSNVQIVRQQSYSIMSVVKEE
bRSV dbj|BAA00798.1         EFSVNAGITTPLSTYMLTNSELLSLINDMPITNDQKKLMSSNVQIVRQQSYSIMSVVKEE
bRSV gb|AAB28458.1          EFSVNAGITTPLSTYMLTNSELLSLINDMPITNDQKKLMSSNVQIVRQQSYSIMSVVKEE
bRSV gb|AAA42808.1          EFSVNAGITTPLSTYMLTNSELLSLINDMPITNDQKKLMSSNVQIVRQQSYSIMSVVKEE
bRSV ref|NP_048055.1        EFSVNAGITTPLSTYMLTNSELLSLINDMPITNDQKKLMSSNVQIVRQQSYSIMSVVKEE
bRSV gb|AAL49399.1          EFSVNAGITTPLSTYMLTNSELLSLINDMPITNDQKKLMSSNVQIVRQQSYSIMSVVKEE
bRSV emb|CAA76980.1         EFSVNAGITTPLSTYMLTNSELLSLINDMPITNDQKKLMSSNVQIVRQQSYSIMSVVKEE
                            *******:***:*************:**************   ************::: *
```

Supplementary Figure 1. Clustal Omega sequence alignment of hRSV F strain A2 with RSV F from nine bovine strains. Each row covers 60 positions. Residue positions completely conserved are designated by a “*”, homologous residues by a “:” and variable residues by a space.

```
hRSV A2 gb|AAB86664.1        VLAYVVQLPLYGVIDTPCWKLHTSPLCTTNTKEGSNICLTRTDRGWYCDNAGSVSFFPQA
bRSV gb|AAA42804.1           VIAYVVQLPLYGVIDTPCWKLHTSPLCTTDNKEGSNICLTRTDRGWYCDNAGSVSFFPQA
bRSV gb|ACL80037.1           VIAYVVQLPIYGVIDTPCWKVHTSPLCTTDNKEGSNICLTRTDRGWYCDNAGSVSFFPQA
bRSV emb|CAN90052.1          VMAYVVQLPIYGVIDTPCWKLHTSPLCTTDNKEGSNICLTRTDRGWYCDNAGSVSFFPQA
bRSV dbj|BAA00798.1          VMAYVVQLPIYGVIDTPCWKLHTSPLCTTDNKEGSNICLTRTDRGWYCDNAGSVSFFPQA
bRSV gb|AAB28458.1           VIAYEVQLPIYGVIDTPCWKIHTSPLCTTDNKEGSNICLTRTDRGWYCDNAGSVSFFPQA
bRSV gb|AAA42808.1           VIAYVVQLPIYGVIDTPCWKLHTSPLCTTDNKEGSNICLTRTDRGWYCDNAGSVSFFPQA
bRSV ref|NP_048055.1         VIAYVVQLPIYGVIDTPCWKLHTSPLCTTDNKEGSNICLTRTDRGWYCDNAGSVSFFPQT
bRSV gb|AAL49399.1           VIAYVVQLPIYGVIDTPCWKLHTSPLCTTDNKEGSNICLTRTDRGWYCDNAGSVSFFPQT
bRSV emb|CAA76980.1          VIAYVVQLPIYGVIDTPCWKLHTSPLCTTDNKEGSNICLTRTDRGWYCDNAGSVSFFPQA
                             *:** ****:**********:********:.****************************:

hRSV A2 gb|AAB86664.1        ETCKVQSNRVFCDTMNSLTLPSEVNLCNVDIFNPKYDCKIMTSKTDVSSSVITSLGAIVS
bRSV gb|AAA42804.1           ETCKVQSNRVFCDTMNSLTLPTDVNLCNTDIFNSKYDCKIMTSKTDISSSVITSIGAIVS
bRSV gb|ACL80037.1           ETCKVQSNRVFCDTMNSLTLPTDVNLCNTDIFNTKYDCKIMTSKTDISSSVITSIGAIVS
bRSV emb|CAN90052.1          ETCKVQSNRVFCDTMNSLTLPTDVNLCNTDIFNAKYDCKIMTSKTDISSSVITSIGAIVS
bRSV dbj|BAA00798.1          ETCKVQSNRVFCDTMNSLTLPTDVNLCNTDIFNAKYDCKIMTSKTDISSSVITSIGAIVS
bRSV gb|AAB28458.1           ETCKVQSNRVFCDTMNSLTLPTDVNLCNTDIFNTKYDCKIMTSKTDISSSVITSIGAIVS
bRSV gb|AAA42808.1           ETCKVQSNRVFCDTMNSLTLPTDVNLCNTDIFNTKYDCKIMTSKTDISSSVITSIGAIVS
bRSV ref|NP_048055.1         ETCKVQSNRVFCDTMNSLTLPTDVNLCNTDIFNTKYDCKIMTSKTDISSSVITSIGAIVS
bRSV gb|AAL49399.1           ETCKVQSNRVFCDTMNSLTLPTDVNLCNTDIFNTKYDCKIMTSKTDISSSVITSIGAIVS
bRSV emb|CAA76980.1          ETCKVQSNRVFCDTMNSLTLPTDVNLCNTDIFNTKYDCKIMTSKTDISSSVITSIGAIVS
                             *********************::*****.**** ************:*******:*****

hRSV A2 gb|AAB86664.1        CYGKTKCTASNKNRGIIKTFSNGCDYVSNKGVDTVSVGNTLYYVNKQEGKSLYVKGEPII
bRSV gb|AAA42804.1           CYGKTKCTASNKNRGIIKTFSNGCDYVSNKGVDTVSVGNTLYYVNKLEGKALYIKGEPII
bRSV gb|ACL80037.1           CYGKTKCTASNKNRGIIKTFSNGCDYVSNKGVDTVSVGNTLYYVNKLEGKALYIKGEPII
bRSV emb|CAN90052.1          CYGKTKCTASNKNRGIIKTFSNGCDYVSNRGVDTVSVGNTLYYVNKLEGKALYIKGEPII
bRSV dbj|BAA00798.1          CYGKTKCTASNKNRGIIKTFSNGCDYVSNRGVDTVSVGNTLYYVNKLEGKALYIKGEPII
bRSV gb|AAB28458.1           CYGKTKCTASNKNRGIIKTFPIGCDYVSNKGVDTVSVGNTLYYVNKLEGKALYIKGEPII
bRSV gb|AAA42808.1           CYGKTKCTASNKNRGIIKTFSNGCDYVSNKGVDTVSVGNTLYYVNKLEGKALYIKGEPII
bRSV ref|NP_048055.1         CYGKTKCTASNKNRGIIKTFSNGCDYVSNKGVDTVSVGNTLYYVNKLEGKALYIKGEPII
bRSV gb|AAL49399.1           CYGKTKCTASNKNRGIIKTFSNGCDYVSNKGVDTVSVGNTLYYVNKLEGKALYIKGEPII
bRSV emb|CAA76980.1          CYGKTKCTASNKNRGIIKTFSNGCDYVSNKGVDTVSVGNTLYYVNKLEGKALYIKGEPII
                             ********************  *******:**************** ***:**:******

hRSV A2 gb|AAB86664.1        NFYDPLVFPSDEFDASISQVNEKINQSLAFIRKSDELLHNVNAGKSTINIMITTIIIVII
bRSV gb|AAA42804.1           NYYNPLVFPSDEFDASIAQVNAKINQSLAFIRRSDELLHSVDVGKSTTNVVITTIIIVIV
bRSV gb|ACL80037.1           NYYNPLVFGTYEFDASIAQVNAK-------------------------------------
bRSV emb|CAN90052.1          NYYDPLVFPSDEFDASIAQVNAKINQSLAFIRRSDELLHSVDVGKSTTNVVITTIIIVIV
bRSV dbj|BAA00798.1          NYYDPLVFPSDEFDASIAQVNAKINQSLAFIRRSDELLHSVDVGKSTTNVVITTIIIVIV
bRSV gb|AAB28458.1           NYYDPLVFPSDEFDASIAQVNAKINQSLAFIRRSDELLHSVDVGKSTTNVVITTIIIVIV
bRSV gb|AAA42808.1           NYYDPLVFPSDEFDASIAQVNAKINQSLAFIRRSDELLHSVDVGKSTTNVVITTIIIVIV
bRSV ref|NP_048055.1         NYYDPLVFPSDEFDASIAQVNAKINQSLAFIRRSDELLHSVDVGKSTTNVVITTIIIVIV
bRSV gb|AAL49399.1           NYYDPLVFPSDEFDASIAQVNAKINQSLAFIRRSDELLHSVDVGKSTTNVVITTIIIVIV
bRSV emb|CAA76980.1          NYYDPLVFPSDEFDASIAQVNAKINQSLAFIRRSDELLHSVDVGKSTTNVVITTIIIVIV
                             *:*:**** : ******:*** *

hRSV A2 gb|AAB86664.1        VILLSLIAVGLLLYCKARSTPVTLSKDQLSGINNIAFSN
bRSV gb|AAA42804.1           VVILMLITVGLLFYCKTRSTPIMLGKDQLSSINNLSFSK
bRSV gb|ACL80037.1           ---------------------------------------
bRSV emb|CAN90052.1          VVILMLIAVGLLFYSKTRSTPIMLGKDQLSGINNLSFSK
bRSV dbj|BAA00798.1          VVILMLIAVGLLFYSKTRSTPIMLGKDQLSGINNLSFSK
bRSV gb|AAB28458.1           VVILMLIAVGLLFYCKTRSTPIMLGKDQLSGINNLSFSK
bRSV gb|AAA42808.1           VVILMLIAVGLLFYCKTRSTPIMLGKDQLSGINNLSFSK
bRSV ref|NP_048055.1         VVILMLIAVGLLFYCKTKSTPIMLGKDQLSGINNLSFSK
bRSV gb|AAL49399.1           VVILMLIAVGLLFYCKTKSTPIMLGKDQLSGINNLSFSK
```

Supplementary Figure 1 (continued). Clustal Omega sequence alignment of hRSV F strain A2 with RSV F from nine bovine strains. Each row covers 60 positions. Residues completely conserved are designated by a "*", homologous residues by a ":" and variable residues by a space.

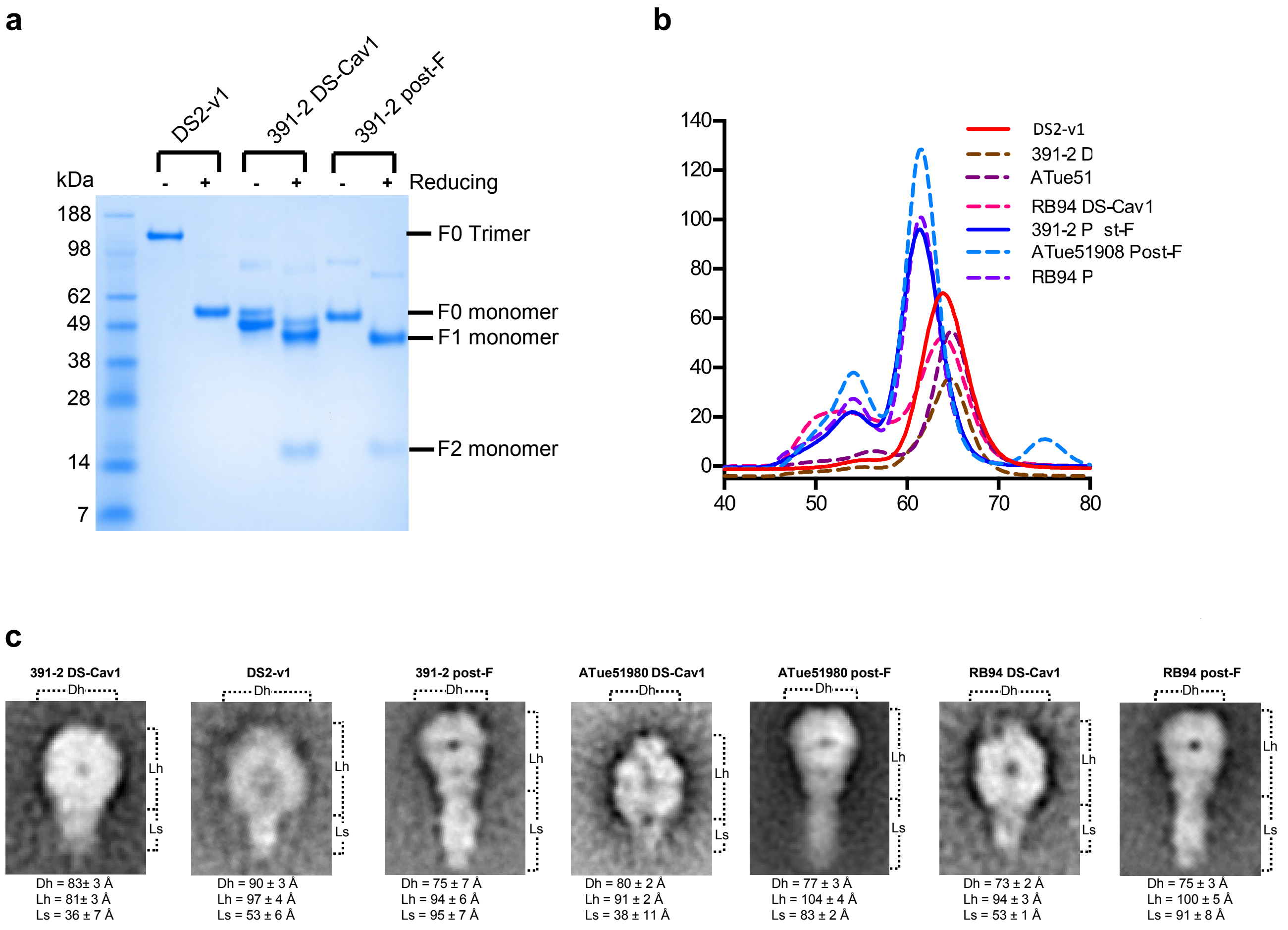

Supplementary Figure 2. Protein purification and electron microscopy analysis. **a,** Representative SDS-PAGE gel analysis for engineered bRSV F glycoproteins. DS2-v1, 391-2 DS-Cav1 and 391-2 post-F. Proteins are observed to collapse to smaller molecular weight bands in the presence of a reducing agent, confirming disulfide bond formation. **b,** Gel filtration chromatograms of bRSV F glycoprotein variants. Variants stabilized in pre-F conformation had longer retention times than variants with post-F conformation. **c,** Negative stain electron microscopy of pre-fusion and post-fusion forms of bovine RSV F. Images shown here are 2D class averages of variants with measured dimensions. Dh, diameter of head; Lh, length of head; Ls, length of stalk.

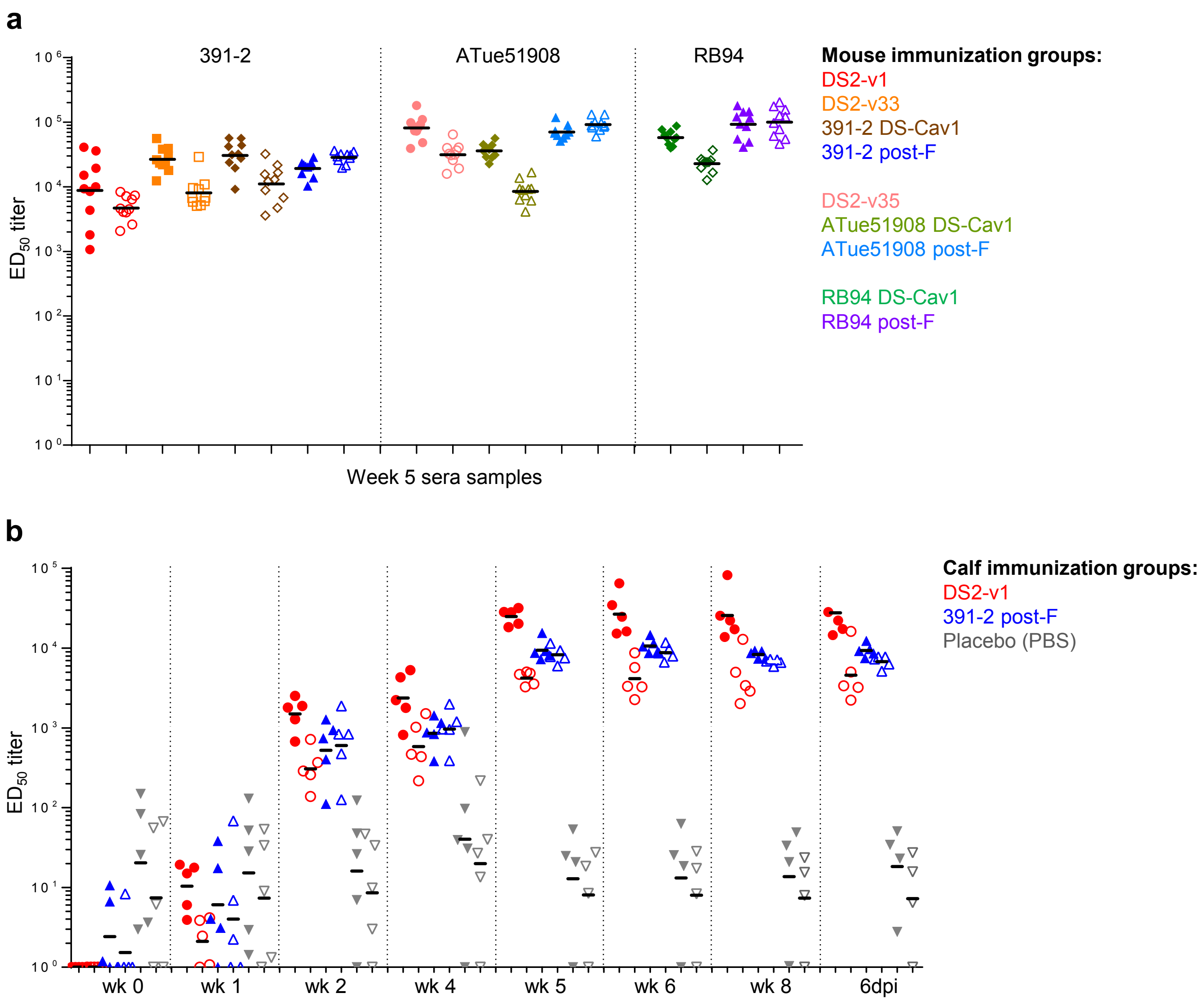

Supplementary Figure 3. Immunogenicity of engineered bovine RSV F pre-F trimers. ELISA binding titers of week five sera from mice **(a)** and longitudinal sera samples from calves **(b)** immunized with bRSV F variants. titers from each animal are represented by color-coded symbols. Solid symbols indicate sera recognition of immobilized DS2-v1 RSV F trimers and open symbols indicate recognition of immobilized 391-2 post-F RSV F trimers. Vertical dotted lines separate immunogen strains in (**a**) and weeks post prime in (**b**). 6dpi, 6 days post inoculation for calf challenge study in (**b**). Geometric mean titers are indicated by black horizontal lines.

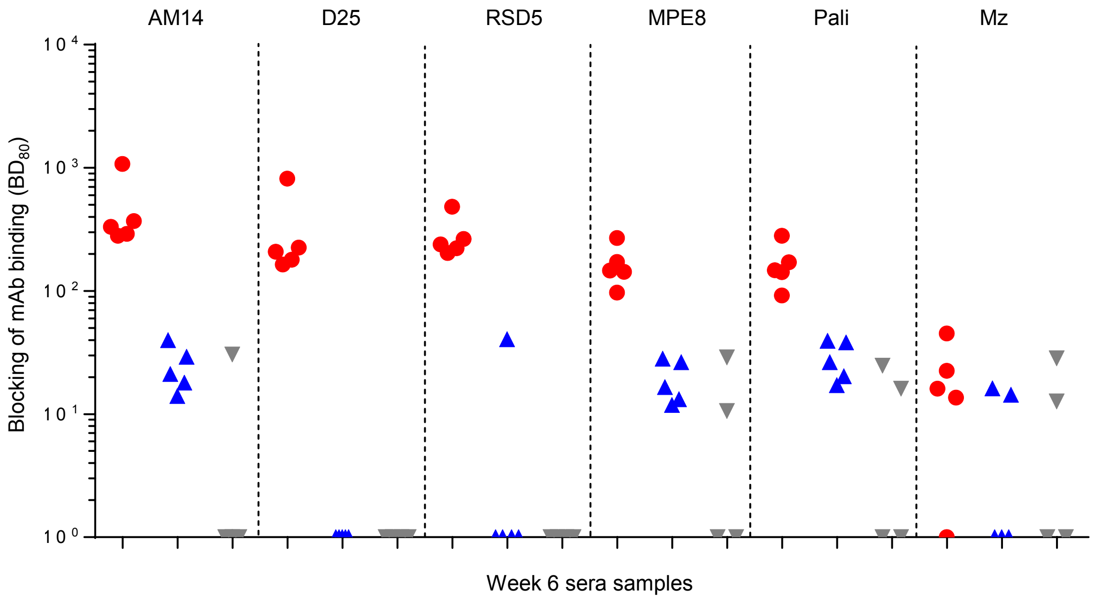

Supplementary Figure 4. Blocking of neutralizing antibody binding. ELISA plates coated with DS2-v1 bRSV F trimer were incubated with serial dilutions of week 6 calf sera followed by biotinylated mAbs. The serum dilution that blocked mAb binding by 80% (defined as BD_{80}) was determined. Higher BD_{80} values indicate the presence of sera that specifically blocks the respective mAbs. Vertical dotted lines separate the six different biotinylated mAbs (indicated above) used for pre-F trimer detection. Color-coding and symbols match that of Supplementary Fig. 3b.

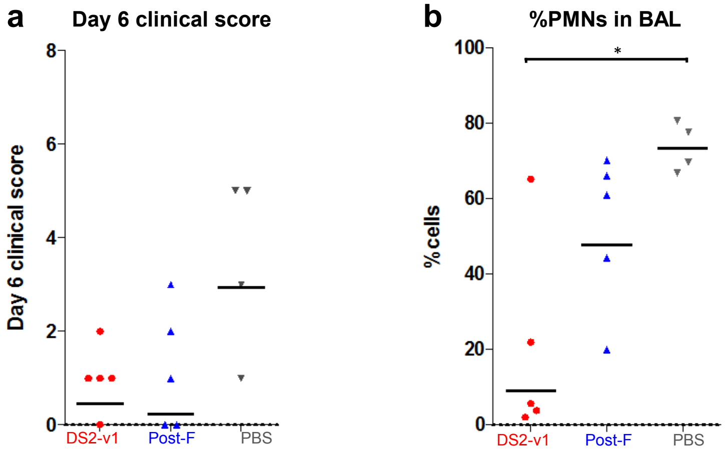

Supplementary Figure 5. Effect of vaccination on clinical signs of disease and neutrophils in BAL. a, Day 6 clinical score. Each dot represents the clinical score of each animal obtained at day 6 post challenge. DS2-v1, Post-F (391-2 post-F), PBS (Placebo). Geometric mean scores are indicated by black horizontal lines. b, Effect of F protein vaccination on proportion of neutrophils in BAL, 6 days after challenge with bRSV. P <0.05 for calves vaccinated with pre-F compared to controls. P values were determined by two-tailed Mann-Whitney tests. * indicates P ≤ 0.05, ** indicates P ≤ 0.01, *** indicates P ≤ 0.001 and **** indicates P ≤ 0.0001.

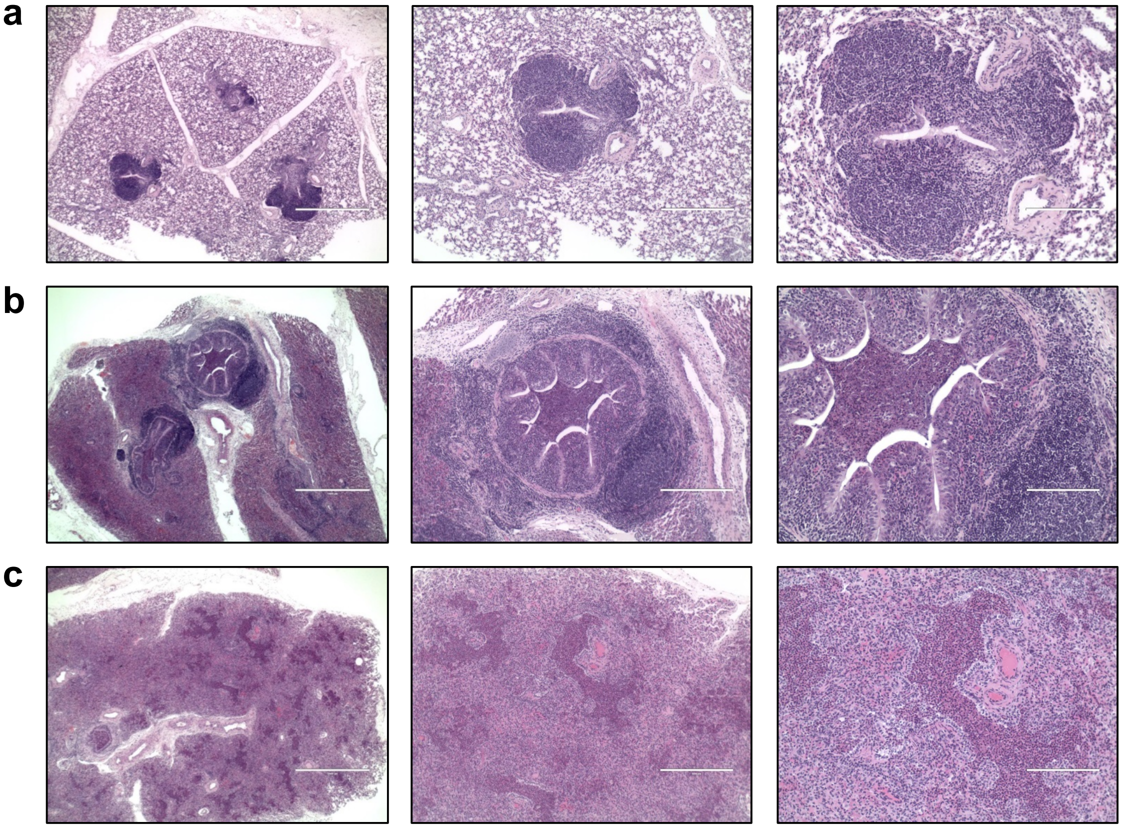

Supplementary Figure 6. Histology of lung sections from vaccinated calves. Haematoxylin and eosin stained lung sections (left panel 4x objective, middle panel 10x objective and right panel 20x objective) from calves vaccinated with 50 µg of adjuvanted pre-F **(a)**, post-F **(b)** and PBS **(c)**, 6 days after challenge with bRSV. White scale bars represent 1000 µm (left panel), 400 µm (middle panel) 200 µm (right panel).
